# COVID-19 Severity and Waning Immunity After up to 4 mRNA Vaccine Doses in 73 608 Patients With Cancer and 621 475 Matched Controls in Singapore: A Nationwide Cohort Study

**DOI:** 10.1001/jamaoncol.2023.2271

**Published:** 2023-07-13

**Authors:** Wei Chong Tan, Janice Yu Jin Tan, Joline Si Jing Lim, Ryan Ying Cong Tan, Ainsley Ryan Yan Bin Lee, Fun Loon Leong, Soo Chin Lee, Louis Yi Ann Chai, Thuan Tong Tan, Muhammad Ismail Bin Abdul Malek, Benjamin Ong, David Chien Lye, Calvin J. Chiew, Wee Joo Chng, Soon Thye Lim, Lavina D. Bharwani, Iain Beehuat Tan, Raghav Sundar, Kelvin Bryan Tan

**Affiliations:** 1 Division of Medical Oncology, National Cancer Centre Singapore, Singapore; 2 Ministry of Health, Singapore; 3 Department of Haematology-Oncology, National University Cancer Institute, Singapore, National University Health System, Singapore; 4 Experimental Therapeutics Programme, Cancer Science Institute, National University of Singapore, Singapore; 5 Yong Loo Lin School of Medicine, National University of Singapore, Singapore; 6 Division of Infectious Diseases, Department of Medicine, National University Health System, Singapore; 7 Department of Medicine, Yong Loo Lin School of Medicine, National University of Singapore, Singapore; 8 Synthetic Biology for Clinical and Technological Innovation, National University of Singapore, Singapore; 9 Department of Infectious Diseases, Singapore General Hospital, Singapore; 10 National Centre for Infectious Diseases, Singapore; 11 Lee Kong Chian School of Medicine, Nanyang Technological University, Singapore; 12 Department of Infectious Diseases, Tan Tock Seng Hospital, Singapore; 13 Department of Medical Oncology, Tan Tock Seng Hospital, Singapore; 14 Genome Institute of Singapore, Singapore; 15 Duke-NUS Medical School, Singapore; 16 Cancer and Stem Cell Biology Program, Duke-NUS Medical School, Singapore; 17 The N.1 Institute for Health, National University of Singapore, Singapore; 18 Singapore Gastric Cancer Consortium, Singapore; 19 Saw Swee Hock School of Public Health, National University of Singapore, Singapore

## Abstract

**Importance:**

Despite patients with cancer being at risk of poor outcomes from COVID-19, there are few published studies for vaccine efficacy in this group, with suboptimal immunogenicity and waning vaccine efficacy described in small studies being a concern.

**Objective:**

To assess the incidence rate of severe COVID-19 disease outcomes associated with the number of vaccine doses received and the waning of protection over time.

**Design, Setting, and Participants:**

A prospective multicenter observational cohort study was carried out over 2 time periods (September 15, 2021, to December 20, 2021 [delta wave], and January 20, 2022, to November 11, 2022 [omicron wave]) predominated by SARS-CoV-2 delta and omicron variants, respectively. Overall, 73 608 patients with cancer (23 217 active treatment, 50 391 cancer survivors) and 621 475 controls matched by age, sex, race and ethnicity, and socioeconomic status were included.

**Exposure:**

Vaccine doses received, from zero to 4 doses, and time elapsed since last vaccine dose.

**Outcomes:**

Competing-risk regression analyses were employed to account for competing risks of death in patients with cancer. Main outcomes were incidence rate ratios (IRRs) of COVID-19 infection, hospitalization, and severe disease (defined as requirement for supplemental oxygen, intensive care, or death). The IRRs stratified by time from last vaccine dose served as indicators of waning of vaccine effectiveness over time.

**Results:**

The mean (SD) age of actively treated patients with cancer, cancer survivors, and controls were 62.7 (14.7), 62.9 (12.6), and 61.8 (14.7) years, respectively. Of 73 608 patients with cancer, 27 170 (36.9%) were men; 60 100 (81.6%) were Chinese, 7432 (10.1%) Malay, 4597 (6.2%) Indian, and 1479 (2.0%) were of other races and ethnicities. The IRRs for the 3-dose and 4-dose vs the 2-dose group (reference) for COVID-19 hospitalization and severe disease were significantly lower during both the delta and omicron waves in cancer and control populations. The IRRs for severe disease in the 3-dose group for active treatment, cancer survivors, and controls were 0.14, 0.13, and 0.07 during the delta wave and 0.29, 0.19, and 0.21 during omicron wave, respectively. The IRRs for severe disease in the 4-dose group during the omicron wave were even lower at 0.13, 0.10 and 0.10, respectively. No waning of vaccine effectiveness against hospitalization and severe disease was seen beyond 5 months after a third dose, nor up to 5 months (the end of this study’s follow-up) after a fourth dose.

**Conclusion:**

This cohort study provides evidence of the clinical effectiveness of mRNA-based vaccines against COVID-19 in patients with cancer. Longevity of immunity in preventing severe COVID-19 outcomes in actively treated patients with cancer, cancer survivors, and matched controls was observed at least 5 months after the third or fourth dose.

## Key Points

**Question:**

How effective is COVID-19 vaccination in patients with cancer in protecting against severe disease and does protection wane over time?

**Findings:**

In this nationwide cohort study including 73 608 patients with cancer and 621 475 matched controls, third and fourth vaccine doses were associated with incremental protection in both groups. No significant decrease in protection against severe disease was observed 5 months after the third and fourth vaccine dose.

**Meaning:**

Each booster COVID-19 vaccine dose was associated with significant clinical protection in patients with cancer, lasting at least 5 months in actively treated patients with cancer and cancer survivors, underscoring the benefit of boosters.

## Teaser

This cohort study examines the incidence rate of severe COVID-19 disease outcomes associated with the number of vaccine doses received and the waning of protection over time.

## Introduction

More than 500 million cases of COVID-19 have been confirmed worldwide^1^ since it was declared as a pandemic by the World Health Organization on March 11, 2020.^2^ Effective vaccines for COVID-19, mainly using the viral surface spike protein as an antigenic target,^3,4^ have been especially important for immunocompromised patients including patients with cancer, who are at increased risk of severe disease and poor outcomes from COVID-19.^5,6,7,8^ However, evidence of suboptimal immunogenicity and waning immunity following vaccination in immunocompromised patients^9,10,11,12^ has prompted the urgent need for booster vaccination especially in this vulnerable population.

Various retrospective studies confirmed the clinical efficacy of COVID-19 vaccination in reducing rates of symptomatic infection and severe disease.^13,14,15^ Although the clinical effectiveness of a third vaccine dose has been demonstrated in the general population,^16^ data in immunocompromised patients have been limited to small studies of postvaccination seroconversion, both for an initial 2-dose series^17,18^ as well as a third dose.^19,20,21,22,23,24^ Furthermore, there is a paucity of evidence of the time course of waning immunity in both immunocompromised and healthy individuals to guide optimal intervals for booster vaccination. As the COVID-19 pandemic progresses, representative data of waning immunity is crucial to guide policy in protecting the general population and vulnerable subgroups.

In Singapore, patients with cancer were first excluded from the initial rollout of vaccines to the general public from February 2021^25^ as part of national vaccination efforts based on safety considerations, but were subsequently made eligible from July 2021 (eFigure 1 in Supplement 1).^26^ Following the implementation of a third vaccine dose, patients with cancer on active treatment were prioritized to receive it as early as 2 months following the second dose, compared with the eligibility of the general public to receive it only after 5 months.^27^ More recently, up to a fifth dose has been made available to the general population, with vulnerable subgroups of patients including those immunocompromised through cancer treatment being prioritized. Four vaccines are currently approved for use in Singapore^28^ based on efficacy demonstrated in randomized clinical trials; the mRNA-based BNT162b2 Comirnaty (Pfizer-BioNTech)^29^ and mRNA-1273 Spikevax (Moderna)^30^ vaccines, protein subunit-based vaccine NVX-CoV2373 Nuvaxovid (Novavax),^31^ and inactivated virus-based vaccine CoronaVac (Sinovac).^32^

To address the limited studies of the clinical effectiveness of vaccines among patients with cancer, this article assesses vaccine effectiveness and waning of effectiveness of up to 4 doses of COVID-19 vaccines among a nation wide sample of patients with cancer compared with the general population.

## Methods

### Setting and Study Population

This multicenter cohort study was conducted by Singapore’s Ministry of Health, and includes all patients on follow-up in the public health care system for a diagnosis of cancer across the 3 tertiary cancer institutions in Singapore; the National Cancer Centre Singapore (NCCS), National University Cancer Institute, Singapore (NCIS), and Tan Tock Seng Hospital (TTSH). Most patients received the mRNA-based vaccines BNT162b2 and mRNA-1273 administered in national vaccination centers in the community and health care institutions. Patients with COVID-19 acquired outside Singapore or prior to the study period were excluded to improve comparability between the study and control populations.

### Study Design

This study was conducted to support policy decision-making and evaluation of public health responses to COVID-19 under the Infectious Disease Act and was exempted from ethics review and written informed consent. Relevant parameters were extracted from national database records maintained as part of Singapore’s national pandemic response efforts. Patient characteristics were collected prospectively and outcomes of COVID-19 infection were predefined prior to commencement of this study; these include baseline demographics (age, sex, race and ethnicity, and socioeconomic status as indicated by residence), cancer treatment at the time of study, number and dates of mRNA-based COVID-19 vaccine doses received, COVID-19 infection and date of infection, and COVID-19–associated hospitalization, requirement for supplemental oxygen, requirement for intensive care, and death. Patients administered zero or 1 vaccine dose were considered as “unvaccinated/partially vaccinated,” 2 doses as “fully vaccinated,” and 3 or 4 doses as “boosted.”

Our analysis was further stratified according to the prevailing COVID-19 variant and time periods of infection waves in Singapore, determined retrospectively. The study population included patients with cancer over 2 separate time periods; September 15, 2021, to December 20, 2021, corresponding to the wave of infections predominated by the SARS-CoV-2 delta variant (delta wave), and January 20, 2022, to November 11, 2022, predominated by the SARS-CoV-2 omicron variant (omicron wave). For each study period, data collected was locked after the last day stipulated, December 15, 2021, for the delta wave and November 11, 2022, for the omicron wave. Five controls from the general population matched by age, sex, race and ethnicity, and socioeconomic status (based on type of residence) from national database records were included for every patient in the study population for comparison.

Vaccination status was categorized according to the number of vaccine doses administered; zero/single dose, 2 doses, 3 doses, and 4 doses. In the cancer cohort, patients receiving active treatment (chemotherapy, immunotherapy, targeted therapy) and no treatment (cancer survivors) were analyzed separately. Incidence of COVID-19 infections and vaccination rates in the study population were compared with the general population. Incidence rate ratios (IRRs) for COVID-19 infection, hospitalizations, and severe disease (defined as requirement for supplemental oxygen, intensive care, or death) by vaccination status were calculated for the cancer cohort and matched controls, as were IRRs over time for fully vaccinated and boosted patients as indicators of waning vaccine effectiveness. Patients who received non–mRNA-based vaccines were excluded from these analyses. Cases and controls were categorized by time elapsed since the last vaccine dose (8-59 days, 60-149 days, and more than 150 days) for analysis on waning vaccine effectiveness. For calculations of IRRs to assess statistical significance, 95% confidence intervals are reported.

### Statistical Analysis

Using methods previously described by Bar-On et al^16^ and with further reference to Zhang et al,^33^ competing-risk regressions taking into account competing risks of non–COVID-19-related deaths were performed to estimate relative risks for comparison across vaccination status categories. Analyses were set up on a person-day basis, adjusted for the covariates of age, sex, race and ethnicity, prior infection with COVID-19 before the study period, and socioeconomic status. Weekly dummy date variables were included to control for varying force of infection across time. Exponentiated coefficients of competing-risks regression were derived and IRRs calculated, with lower IRR values indicating better vaccine efficacy compared with designated reference groups. Fully vaccinated patients were the reference for comparisons of vaccine effectiveness across patients of different vaccination status. Poisson regressions controlling for the same covariates were conducted for comparison.

To further improve the robustness of results, we conducted calendar time-scale Cox regressions following Lund et al,^34^ with and without accounting for competing risks of non–COVID-19 deaths. Similar covariates were included in these Cox regressions. For waning of vaccine effectiveness, fully vaccinated patients within 8 to 59 days were the reference group in the delta phase, whereas boosted patients within 8 to 59 days were the reference group in the omicron phase for better comparability. The IRRs for patients were grouped by number of vaccine doses received (zero/single-dose, 2-dose, 3-dose, and 4-dose groups), with lower IRRs being reflective of decreased incidence of patients with corresponding COVID-19–related outcomes of infection, severe disease, or hospitalization.

Data analysis was carried out in STATA statistical software (StataCorp LP, version 17). *P*<.05 was considered statistically significant.

## Results

### Characteristics and Vaccination Rates in the Study Population

Overall, 73 608 individual patients with cancer (23 217 receiving active treatment and 50 391 cancer survivors) with 621 475 matched controls from the general population were included across the delta and omicron waves (baseline characteristics shown in Table 1). Cumulative incidence of COVID-19 infections among actively treated patients with cancer, cancer survivors, and matched controls over delta and omicron waves is shown in eFigure 2 in Supplement 1. The total observed person-days and incidence rate per million person-days is shown in eTable 1 in Supplement 1. In the general population, 84.8% received at least 2 doses of mRNA-based vaccine by the end of the study period. There were 2.6% who received non–mRNA-based vaccines, and are excluded from comparative analyses focusing on mRNA-based vaccination rates in subsequent sections of this study. Rates of vaccination across the delta and omicron waves are reflected in Table 2.

**Table 1.** Characteristics of Study and Control Populations

Characteristic	0/1 Doses			2 Doses			3 Doses			4 Doses		
	Active treatment	Cancer survivors	Controls	Active treatment	Cancer survivors	Controls	Active treatment	Cancer survivors	Controls	Active treatment	Cancer survivors	Controls
Sex, No. (%)												
Female	727 (61.8)	927 (55.5)	18 865 (52.5)	2286 (59.9)	2949 (56.8)	32 857 (58.7)	9303 (67.4)	20 142 (64.5)	257 162 (65.5)	2907 (65.7)	7197 (58.5)	81 765 (59.7)
Male	450 (38.2)	743 (44.5)	17 095 (47.5)	1533 (40.1)	2244 (43.2)	23 124 (41.3)	4496 (32.6)	11 079 (35.5)	135 398 (34.5)	1515 (34.3)	5110 (41.5)	55 209 (40.3)
Age, y, No. (%)												
0-17	6 (0.5)	34 (2.0)	280 (0.8)	6 (0.2)	38 (0.7)	307 (0.5)	5 (0)	56 (0.2)	688 (0.2)	0	4 (0)	3 (0)
18-29	10 (0.8)	25 (1.5)	266 (0.7)	67 (1.8)	149 (2.9)	1956 (3.5)	211 (1.5)	1070 (3.4)	12 484 (3.2)	10 (0.2)	10 (0.1)	74 (0.1)
30-39	31 (2.6)	60 (3.6)	743 (2.1)	133 (3.5)	320 (6.2)	3526 (6.3)	526 (3.8)	1936 (6.2)	24 027 (6.1)	25 (0.6)	21 (0.2)	208 (0.2)
40-49	95 (8.1)	150 (9.0)	2487 (6.9)	361 (9.5)	567 (10.9)	6811 (12.2)	1700 (12.3)	4447 (14.2)	58 153 (14.8)	91 (2.1)	178 (1.4)	1843 (1.3)
50-59	234 (19.9)	241 (14.4)	5944 (16.5)	784 (20.5)	903 (17.4)	12 788 (22.8)	3175 (23.0)	6505 (20.8)	86 511 (22.0)	790 (17.9)	1895 (15.4)	23 327 (17.0)
60-69	407 (34.6)	372 (22.3)	10 745 (29.9)	1216 (31.8)	1245 (24.0)	14 238 (25.4)	4353 (31.5)	8282 (26.5)	109 174 (27.8)	1627 (36.8)	4077 (33.1)	47 092 (34.4)
70-79	278 (23.6)	454 (27.2)	9046 (25.2)	916 (24.0)	1222 (23.5)	10 031 (17.9)	2986 (21.6)	6309 (20.2)	73 686 (18.8)	1331 (30.1)	3865 (31.4)	41 643 (30.4)
≥80	116 (9.9)	334 (20.0)	6449 (17.9)	336 (8.8)	749 (14.4)	6324 (11.3)	843 (6.1)	2616 (8.4)	27 837 (7.1)	548 (12.4)	2257 (18.3)	22 784 (16.6)
Race and ethnicity, No. (%)												
Chinese	923 (78.4)	1300 (77.8)	27 777 (77.2)	2980 (78.0)	4161 (80.1)	43 042 (76.9)	10 908 (79.0)	25 316 (81.1)	314 693 (80.2)	3744 (84.7)	10 768 (87.5)	118 474 (86.5)
Malay	152 (12.9)	165 (9.9)	4179 (11.6)	503 (13.2)	585 (11.3)	7805 (13.9)	1684 (12.2)	3204 (10.3)	43 443 (11.1)	354 (8.0)	785 (6.4)	9731 (7.1)
Indian	81 (6.9)	160 (9.6)	3114 (8.7)	256 (6.7)	374 (7.2)	4356 (7.8)	907 (6.6)	2076 (6.6)	26 485 (6.7)	208 (4.7)	535 (4.3)	6067 (4.4)
Others	21 (1.8)	45 (2.7)	890 (2.5)	80 (2.1)	73 (1.4)	778 (1.4)	300 (2.2)	625 (2.0)	7939 (2.0)	116 (2.6)	219 (1.8)	2702 (2.0)
Residence, No. (%)												
Public (1-3 room)^a^	395 (33.6)	556 (33.3)	11 798 (32.8)	1111 (29.1)	1541 (29.7)	17 407 (31.1)	3305 (24.0)	7172 (23.0)	90 354 (23.0)	983 (22.2)	2938 (23.9)	32 534 (23.8)
Public (4-5 room)^a^	614 (52.2)	810 (48.5)	18 049 (50.2)	2172 (56.9)	2727 (52.5)	31 166 (55.7)	8209 (59.5)	17 738 (56.8)	226 921 (57.8)	2388 (54.0)	6335 (51.5)	71 684 (52.3)
Private^a^	133 (11.3)	235 (14.1)	4968 (13.8)	431 (11.3)	740 (14.2)	5728 (10.2)	1914 (13.9)	5402 (17.3)	63 724 (16.2)	936 (21.2)	2735 (22.2)	29 533 (21.6)
Others	35 (3.0)	69 (4.1)	1145 (3.2)	105 (2.7)	185 (3.6)	1680 (3.0)	371 (2.7)	909 (2.9)	11 561 (2.9)	115 (2.6)	299 (2.4)	3223 (2.4)
COVID-19, No. (%)												
Infected^b^	453 (38.5)	684 (41.0)	3681 (10.2)	2287 (59.9)	3380 (65.1)	41 815 (74.7)	5977 (43.3)	14 428 (46.2)	164 212 (41.8)	532 (12.0)	1697 (13.8)	15 725 (11.5)
Hospitalized	253 (21.5)	354 (21.2)	1492 (4.1)	705 (18.5)	699 (13.5)	4919 (8.8)	888 (6.4)	993 (3.2)	6246 (1.6)	67 (1.5)	144 (1.2)	992 (0.7)
Severe disease^c^	67 (5.7)	145 (8.7)	646 (1.8)	105 (2.7)	171 (3.3)	1162 (2.1)	100 (0.7)	183 (0.6)	1128 (0.3)	9 (0.2)	23 (0.2)	147 (0.1)
Death^d^	22 (1.9)	53 (3.2)	241 (0.7)	23 (0.6)	35 (0.7)	242 (0.4)	13 (0.1)	21 (0.1)	128 (0)	0	3 (0)	18 (0)
Total, No. (%)	1177 (100)	1670 (100)	35 960 (100)	3819 (100)	5193 (100)	55 981 (100)	13 799 (100)	31 221 (100)	392 560 (100)	4422 (100)	12 307 (100)	136 974 (100)

^a^ Type of residence was used as an indicator of socioeconomic status.

^b^ Significant differences were observed for incidence of COVID-19 infection between the active treatment (*P* < .001) and cancer survivors (*P* < .001) groups compared with controls.

^c^ Significant differences were observed for incidence of severe disease between those in the active treatment (*P* < .001) and cancer survivor (*P* < .001) groups compared with controls. Severe disease was defined as COVID-19 infection with resulting need for supplemental oxygen, intensive care admission, or death.

^d^ Significant differences were observed for death from COVID-19 between the active treatment (*P* < .001) and cancer survivors (*P* < .001) groups compared with controls.

**Table 2.** Vaccination Rates in Patients With Cancer and the General Population

Vaccine doses received	Start of delta wave (September 15, 2021), No. (%)			End of delta wave (December 20, 2021), No. (%)		
	Patients with cancer		General population	Patients with cancer		General population
	Active treatment	Cancer survivors		Active treatment	Cancer survivors	
0 doses	3527 (15.2)	4258 (8.4)	885 274 (21.1)	1030 (4.4)	1487 (3.0)	762 264 (18.2)
1 dose (mRNA-based)	2273 (9.8)	1973 (3.9)	91 946 (2.2)	384 (1.7)	398 (0.8)	15 615 (0.4)
2 doses (mRNA-based)	17 212 (74.1)	43 559 (86.4)	3 152 609 (75.3)	11 874 (51.1)	18 959 (37.6)	1 910 481 (45.6)
3 doses (mRNA-based)	3 (0)	5 (0)	204 (0)	9283 (40.0)	28 020 (55.6)	1 393 995 (33.3)
4 doses (mRNA-based)	0	0	0	0	0	77 (0)
Non-mRNA	203 (0.9)	596 (1.2)	59 186 (1.4)	647 (2.8)	1527 (3.0)	106 787 (2.5)
Total	23 217 (100)	50 391 (100)	4 189 219 (100)	23 217 (100)	50 391 (100)	4 189 219 (100)
	Start of omicron wave (January 20, 2022), No. (%)			End of omicron wave (November 11, 2022), No. (%)		
0 doses	921 (4.0)	1334 (2.6)	666 187 (15.9)	721 (3.1)	1102 (2.2)	492 199 (11.7)
1 dose (mRNA-based)	263 (1.1)	282 (0.6)	97 341 (2.3)	203 (0.9)	189 (0.4)	33 865 (0.8)
2 doses (mRNA-based)	9323 (40.2)	12 979 (25.8)	1 244 467 (29.7)	2206 (9.5)	2293 (4.6)	318 529 (7.6)
3 doses (mRNA-based)	12 033 (51.8)	34 196 (67.9)	2 070 688 (49.4)	13 467 (58.0)	28 749 (57.1)	2 687 293 (64.1)
4 doses (mRNA-based)	0	0	105 (0)	5729 (24.7)	15 908 (31.6)	522 294 (12.5)
Non-mRNA	678 (2.9)	1600 (3.2)	110 431 (2.6)	891 (3.8)	2150 (4.3)	135 039 (3.2)
Total	23 217 (100)	50 391 (100)	4 189 219 (100)	23 217 (100)	50 391 (100)	4 189 219 (100)

At the start of the delta wave, more cancer survivors (86.5%, *P* < .001) and slightly less of those receiving active treatment (74.1%, *P* < .001) were fully vaccinated or boosted compared with the general population (75.3%). As national policy prioritized vaccinations and boosters for immunocompromised patients, increasing numbers receiving active cancer treatment (91.1%, *P* < .001) and cancer survivors (93.2%, *P* < .001) were fully vaccinated or boosted compared with the general population (78.8%) by the end of the delta wave on December 20, 2021. Moving into the omicron wave, the proportion of fully vaccinated patients continued to remain significantly higher in the active treatment (92.0%, *P* < .001) and cancer survivors (93.6%, *P* < .001) cohorts compared with the general population (79.1%).

Booster fourth doses were made available in the later half of the omicron wave. By the end of the omicron wave on November 11, 2022, significantly higher proportions of patients in the active cancer (24.7%) and cancer survivor (31.6%) groups had received a fourth dose compared with the general population (12.5%, *P* < .001). As of the end of this study, 401 (0.54%) of the patients with cancer and 0.15% of the general population had received a fifth vaccine dose, with the numbers too small for any significant analyses to be performed.

### Incidence Rate Ratios

The IRRs for patients grouped by number of vaccine doses received (zero/single-dose, 2-dose, 3-dose, and 4-dose groups) are shown in Table 3. During both the delta and omicron waves, IRRs for COVID-19 infection were not significantly higher in the zero/single-dose group in active treatment, cancer survivors, and control cohorts compared with the 2-dose group. This is likely owing to nationwide implementation of vaccination-differentiated safe management measures, which imposed significant restrictions on social activities for unvaccinated or partially vaccinated (zero/single-dose) patients in an effort to limit transmission of COVID-19. The IRRs for COVID-19 hospitalization in the zero/single-dose group are not shown because its interpretation is confounded by national health care protocols mandating inpatient treatment of unvaccinated or partially vaccinated patients with confirmed COVID-19 infection regardless of disease severity.

**Table 3.** Incidence and Risk of COVID-19 by Unvaccinated or Partially Vaccinated, Fully Vaccinated, and Boosted Populations

Vaccine doses	COVID-19 incidence (95% CI)			COVID-19 hospitalization (95% CI)			COVID-19 severe disease (95% CI)		
	Active treatment	Cancer survivors	Controls	Active treatment	Cancer survivors	Controls	Active treatment	Cancer survivors	Controls
**Delta wave**									
0 or 1 dose	0.64 (0.50-0.82)	0.92 (0.75-1.13)	0.28 (0.26-0.30)	1.57 (1.18-2.10)	2.80 (2.16-3.62)	0.99 (0.88-1.11)	3.37 (1.92-5.91)	9.37 (6.34-13.84)	2.51 (2.12-2.96)
2 doses	1 [Reference]	1 [Reference]	1 [Reference]	1 [Reference]	1 [Reference]	1 [Reference]	1 [Reference]	1 [Reference]	1 [Reference]
3 doses	0.37 (0.29-0.48)	0.34 (0.29-0.40)	0.26 (0.25-0.28)	0.24 (0.15-0.38)	0.23 (0.16-0.33)	0.14 (0.11-0.17)	0.14 (0.03-0.59)	0.13 (0.05-0.33)	0.07 (0.05-0.12)
**Omicron wave**									
0 or 1 dose	0.71 (0.59-0.87)	0.73 (0.63-0.85)	0.40 (0.37-0.44)	NA^a^	NA^a^	NA^a^	1.85 (0.99-3.47)	1.56 (1.02-2.39)	0.82 (0.62-1.08)
2 doses	1 [Reference]	1 [Reference]	1 [Reference]	1 [Reference]	1 [Reference]	1 [Reference]	1 [Reference]	1 [Reference]	1 [Reference]
3 doses	0.91 (0.86-0.97)	0.91 (0.86-0.96)	0.91 (0.88-0.93)	0.45 (0.40-0.52)	0.27 (0.24-0.32)	0.29 (0.27-0.32)	0.29 (0.20-0.42)	0.19 (0.14-0.26)	0.21 (0.18-0.25)
4 doses	0.78 (0.70-0.88)	0.80 (0.74-0.87)	0.85 (0.81-0.88)	0.24 (0.18-0.33)	0.15 (0.12-0.19)	0.21 (0.18-0.25)	0.13 (0.06-0.31)	0.10 (0.06-0.17)	0.10 (0.07-0.15)

^a^ Incidence ratio ratios for COVID-19 hospitalization not shown because interpretation was confounded by mandatory inpatient treatment of unvaccinated or partially vaccinated patients with confirmed COVID-19 infection.

Despite lower COVID-19 infection rates for the zero/single-dose compared with the 2-dose group, these patients had significantly higher rates of severe COVID-19 disease during the delta wave in both the active treatment (IRR, 3.86) and cancer survivor (IRR, 10.07) groups. In addition, the IRR for severe COVID-19 disease for the zero/single-dose group during the omicron wave was also higher in both the active treatment (1.85) and cancer survivor cohorts (1.56), although this did not reach statistical significance in the active treatment cohort. This highlights the increased vulnerability of patients with cancer to adverse outcomes from COVID-19, with risk of severe disease further compounded in the absence of vaccination.

The 3-dose group was significantly protected from COVID-19 hospitalization and severe disease in both cancer and control groups, with lower IRRs compared with the 2-dose group across both the delta and omicron waves. Compared with controls, higher IRR was observed against severe COVID-19 disease in the active treatment cohort during both the delta (IRR, 0.14 vs 0.07) and omicron (IRR, 0.27 vs 0.18) waves, whereas the cancer survivor cohort had similar IRRs to controls (IRR, 0.13 vs 0.07 during the delta wave, 0.18 vs 0.18 during the omicron wave).

During the omicron wave, numerically lower IRRs against severe disease in the 4-dose compared with the 3-dose groups were suggestive of additional protection conferred by a fourth dose. In particular, the IRR for severe disease was significantly lower during infection 8 to 59 days after the fourth dose compared with 8 to 59 days after the third dose (eTable 2 in Supplement 1), suggesting a fourth dose provides additional protection.

For the outcome of COVID-19 hospitalization, IRRs were significantly lower in the 4-dose vs the 3-dose group in the active treatment (0.24 vs 0.45), cancer survivor (0.15 vs 0.27), and control groups (0.21 vs 0.29), suggesting a fourth vaccine dose may confer additional protection against COVID-19 infection necessitating hospitalization, regardless of cancer and treatment status.

### Waning of Vaccine Effectiveness

The overall trends in waning of vaccine effectiveness are depicted in the Figure. During the delta wave, no significant waning of vaccine effectiveness following 2 doses was observed in the active treatment and cancer survivor cohorts against COVID-19 hospitalization and severe disease, which is more remarkable given the significant waning demonstrated in the control group from 150 days postvaccination (eTable 2 in Supplement 1). Although no significant waning following third dose was observed, numbers were small during the delta wave, which limits interpretation of results.

**Figure.**
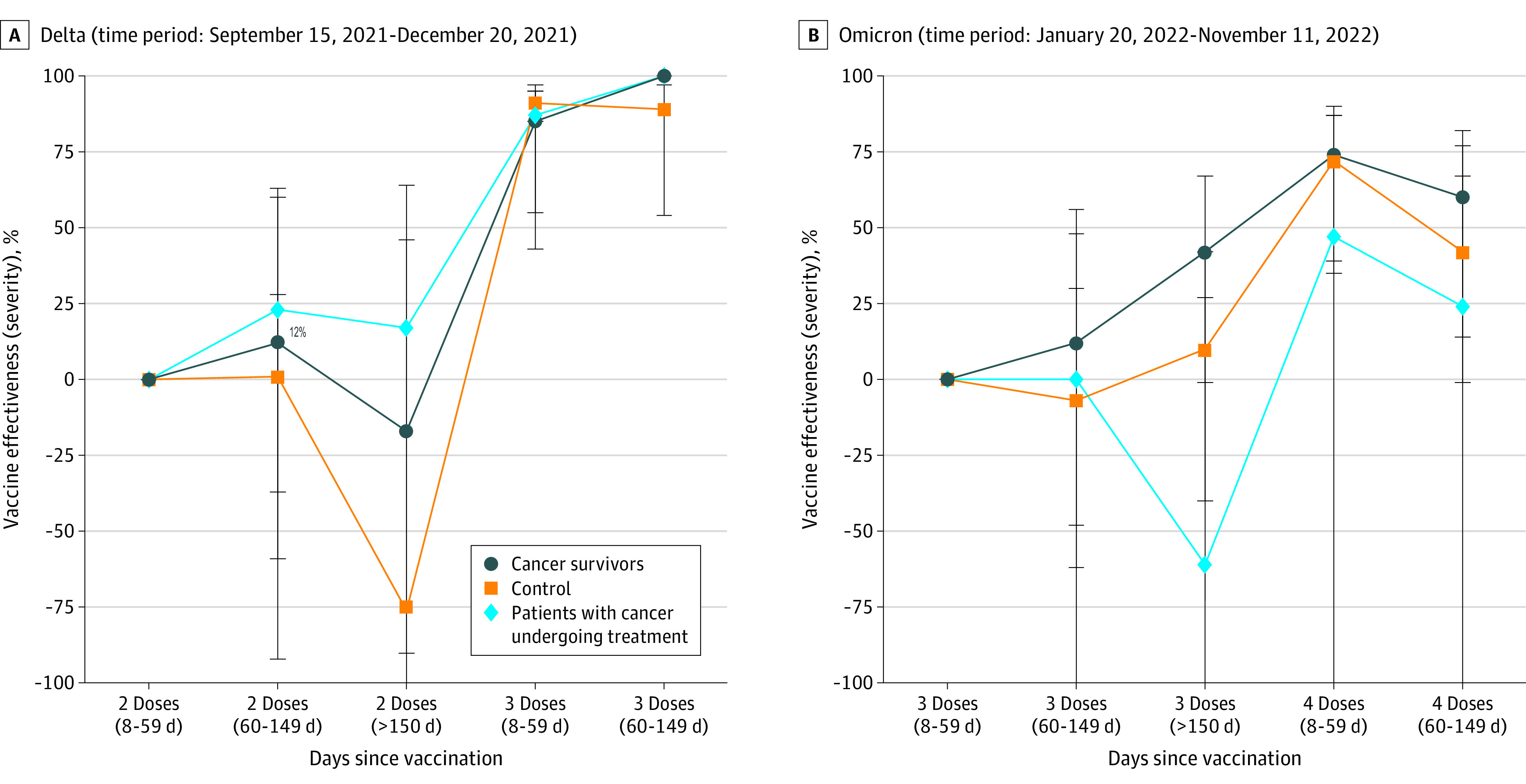
Waning of Vaccine Effectiveness Across the Delta and Omicron Waves A, Shows the delta period; and B, the omicron period for the waning of vaccine effectiveness against severe COVID-19 with reference to 2 doses (8 to 59 days) and 3 doses (8 to 59 days), respectively. No significant waning was observed. The whiskers show the 95% CIs.

During the omicron wave, no significant waning of vaccine effectiveness following 3 doses was observed against COVID-19 hospitalization and severe disease in both the cancer and control cohorts, despite waning effectiveness against COVID-19 infection shown. The incidence of COVID-19 outcomes 8 to 59 days after the third dose was used as the reference.

Following the fourth dose, no significant waning of vaccine effectiveness was observed across all outcomes from 60 days postvaccination. However, small numbers may limit interpretation of these results, given only 0.2% of patients in the 4-dose group manifested the outcome of severe disease.

### Sensitivity and Additional Analyses

Additional analyses, including Poisson regressions (eTables 3, 4, 5, and 6 in Supplement 1), calendar-time scale Cox regressions with and without incorporating competing risks (eTables 7, 8, 9, and 10 in Supplement 1), and regressions using time since last dose as a continuous variable (eTable 11 in Supplement 1) were run and compared with results derived from our primary competing risks regression methodology, demonstrating similar trends.

Adjusted absolute incidence rates per million person-days (eTables 1 and 12 in Supplement 1) were lower during the delta wave for the 3-dose compared with the 2-dose group for COVID-19 severe disease in the active treatment (5 vs 29), cancer survivor (4 vs 21), and control (2 vs 20) groups. The same was observed during the omicron wave for the 4-dose compared with the 3-dose group (active treatment, 21 vs 29; cancer survivors, 17 vs 21), though not in the control group (11 vs 11).

## Discussion

To our knowledge, this study is the first to demonstrate clinical vaccine effectiveness of up to 4 doses of mRNA-based vaccines against COVID-19 in actively treated patients with cancer and cancer survivors, and matched noncancer controls at a population level. In addition, few other studies have longitudinally correlated the time from the last vaccination to clinical outcomes of COVID-19 infection in patients with cancer at a representative population level.^35^

High vaccination rates likely accounted for the low COVID-19 mortality observed among patients with cancer in Singapore reported in our study compared with prevailing case-fatality rates.^36^ Vaccination-differentiated management measures directed toward reducing exposure of unvaccinated and partially vaccinated patients may have resulted in lower infection rates, although higher incidence of severe disease was still observed in unvaccinated and partially vaccinated individuals. This persisted into the omicron wave, despite the variant being associated with decreased severity compared with preceding variant strains.^37,38,39,40,41,42^

The findings of this cohort study provide evidence that patients receiving a third vaccine dose derive additional protection against severe infection from both the delta and omicron variants, consistent with results from previous studies.^16,43,44,45,46,47,48^ Between September 15, 2021, to November 11, 2022, of the total 4 756 102 doses administered to the study population, 4 462 301 (93.8%) were monovalent mRNA vaccines, 76 247 (1.6%) were bivalent mRNA vaccines and the remaining 217 554 (4.6%) were non-mRNA vaccines. Significant risk reduction of severe COVID-19 with a third dose was shown in actively treated patients with cancer, although to a lesser extent compared with matched controls. Patients infected more than 150 days after their third dose did not show significantly higher risk of hospitalization and severe disease compared with those infected before 60 days, suggesting a sustained protective effect. Singapore began administering the fourth dose 5 months following the third, being among the first in the world to do so.^49^ After a fourth vaccine dose, the risk reduction of hospitalization and severe disease during the omicron wave was even greater. This suggests that an approach of boosting at regular intervals may be an effective measure against successive waves of COVID-19.

### Strengths and Limitations

This study was first limited by its design as a cohort study because results may be subject to confounding variables that could not be controlled for. Second, rates of hospitalization could have been confounded by national health care protocols mandating inpatient admission and evaluation for patients with cancer with confirmed COVID-19 infection regardless of disease severity. Third, we were unable to control for individual behaviors affecting seeking of medical care and COVID-19 infection risk. These limitations were addressed by defining severity with the clinical end point of COVID-19 infection requiring supplemental oxygen, intensive care admission, or resulting in death, which may be extrapolated widely to other countries and settings. Finally, we were not able to perform regression analyses according to the time elapsed from last treatment in cancer survivors.

Nevertheless, our study has several strengths. First, the study included all cancer patients receiving follow-up in tertiary public institutions, making the sample highly representative of the population. Second, matched controls from the general population allowed us to better isolate the mediating effect of cancer history and active cancer treatment on COVID-19 infection and severity. In this respect, using competing risk regressions in our methodology accounted for the effects of competing comorbidities, a significant consideration in patients with cancer, without introducing unnecessary bias in the results from censoring patients with non–COVID-19 related adverse outcomes. Third, analysis was based on accurate and comprehensive records collected as part of Singapore’s national pandemic response efforts, mitigating missed outcomes and loss to follow-up. Fourth, as primarily mRNA-based monovalent vaccines were administered to the Singapore population during the study period, there was minimal confounding of vaccine efficacy results due to differential efficacy from different COVID-19 vaccines. Finally, the study reports data on clinical population-based efficacy of a fourth vaccine dose, both in immunocompromised cohorts of patients with cancer with or without active treatment and the general population.

## Conclusions

The results of this cohort study provide evidence of the benefit of early vaccination and administration of booster vaccine doses against COVID-19 in patients with cancer, especially in conferring protective effects of vaccination against adverse outcomes of COVID-19. The findings also provided insight into the longevity of vaccine-mediated protection against clinical infection outcomes in both immunocompromised patients with cancer with or without active treatment and the general population. As the world moves toward living with COVID-19, evidence of the booster third and fourth vaccine dose and efficacy in overcoming waning immunity is imperative to guide the optimum protection for our most vulnerable patients.

## Notes

Supplement 1. eFigure 1. Summary timeline of landmark national COVID-19 vaccine recommendations in Singapore eFigure 2. Cumulative incidence rate of COVID-19 infections in actively-treated cancer patients, cancer survivors and matched controls during Delta and Omicron COVID-19 variant infection waves. eTable 1. Person-Days and Incidence Rate per Million Person-Days eTable 2. Waning of Vaccine Effectiveness across the Delta and Omicron Phases (Competing Risks Person-Days Regression) eTable 3. Incidence and Risk of COVID-19 by Unvaccinated/Partially Vaccinated, Fully Vaccinated and Boosted Populations (Poisson Person-Days Regression) eTable 4. Sensitivity Analysis for Incidence and Risk of COVID-19 Severe Disease by Unvaccinated/Partially Vaccinated, Fully Vaccinated and Boosted Populations (Poisson person-days regression) eTable 5. Waning of Vaccine Effectiveness across the Delta and Omicron Phases (Poisson Person-Days Regressions) eTable 6. Sensitivity Analysis for Waning of Vaccine Effectiveness Against COVID-19 Severe Disease across the Delta and Omicron Phases (Poisson Person-Days Regressions) eTable 7. Incidence and Risk of COVID-19 by Unvaccinated/Partially Vaccinated, Fully Vaccinated and Boosted Populations (Calendar-Time Scale Cox Regression) eTable 8. Incidence and Risk of COVID-19 by Unvaccinated/Partially Vaccinated, Fully Vaccinated and Boosted Populations (Calendar-Time Scale Cox Regression Incorporating Competing Risk) eTable 9. Waning of Vaccine Effectiveness across the Delta and Omicron Phases (Calendar-Time Scale Cox Regression) eTable 10. Waning of Vaccine Effectiveness across the Delta and Omicron Phases (Calendar-Time Scale Cox Regression incorporating Competing Risks) eTable 11. Regressions with Time Since Last Dose as a Continuous Variable eTable 12. Person-Days and Incidence Rate/Million Person-Days by Time from Last Vaccine Dose

Supplement 2. Data Sharing Statement
