# Supplementary material for: COVID-19 Severity and Waning Immunity After up to 4 mRNA Vaccine Doses in 73 608 Patients With Cancer and 621 475 Matched Controls in Singapore: A Nationwide Cohort Study
Source: JAMA Oncol. 2023 Jul 13;9(9):1221–9. doi: 10.1001/jamaoncol.2023.2271 (PMC10346511; doi:10.1001/jamaoncol.2023.2271)
Supplement: Supplement 1. — eFigure 1. Summary timeline of landmark national COVID-19 vaccine recommendations in Singapore eFigure 2. Cumulative incidence rate of COVID-19 infections in actively-treated cancer patients, cancer survivors and matched controls during Delta and Omicron COVID-19 variant infection waves. eTable 1. Person-Days and Incidence Rate per Million Person-Days eTable 2. Waning of Vaccine Effectiveness across the Delta and Omicron Phases (Competing Risks Person-Days Regression) eTable 3. Incidence and Risk of COVID-19 by Unvaccinated/Partially Vaccinated, Fully Vaccinated and Boosted Populations (Poisson Person-Days Regression) eTable 4. Sensitivity Analysis for Incidence and Risk of COVID-19 Severe Disease by Unvaccinated/Partially Vaccinated, Fully Vaccinated and Boosted Populations (Poisson person-days regression) eTable 5. Waning of Vaccine Effectiveness across the Delta and Omicron Phases (Poisson Person-Days Regressions) eTable 6. Sensitivity Analysis for Waning of Vaccine Effectiveness Against COVID-19 Severe Disease across the Delta and Omicron Phases (Poisson Person-Days Regressions) eTable 7. Incidence and Risk of COVID-19 by Unvaccinated/Partially Vaccinated, Fully Vaccinated and Boosted Populations (Calendar-Time Scale Cox Regression) eTable 8. Incidence and Risk of COVID-19 by Unvaccinated/Partially Vaccinated, Fully Vaccinated and Boosted Populations (Calendar-Time Scale Cox Regression Incorporating Competing Risk) eTable 9. Waning of Vaccine Effectiveness across the Delta and Omicron Phases (Calendar-Time Scale Cox Regression) eTable 10. Waning of Vaccine Effectiveness across the Delta and Omicron Phases (Calendar-Time Scale Cox Regression incorporating Competing Risks) eTable 11. Regressions with Time Since Last Dose as a Continuous Variable eTable 12. Person-Days and Incidence Rate/Million Person-Days by Time from Last Vaccine Dose [file jamaoncol-e232271-s001.pdf]

Supplemental Online Content

Tan WC, Tan JYJ, Lim JSJ, et al.. COVID-19 severity and waning immunity after up to 4 mRNA vaccine doses in 73 608 patients with cancer and 621 475 matched controls in Singapore: a nationwide cohort study. *JAMA Oncol*. Published online July 13, 2023. doi:10.1001/jamaoncol.2023.2271

eFigure 1. Summary timeline of landmark national COVID-19 vaccine recommendations in Singapore

eFigure 2. Cumulative incidence rate of COVID-19 infections in actively-treated cancer patients, cancer survivors and matched controls during Delta and Omicron COVID-19 variant infection waves

eTable 1. Person-Days and Incidence Rate per Million Person-Days

eTable 2. Waning of Vaccine Effectiveness across the Delta and Omicron Phases (Competing Risks Person-Days Regression)

eTable 3. Incidence and Risk of COVID-19 by Unvaccinated/Partially Vaccinated, Fully Vaccinated and Boosted Populations (Poisson Person-Days Regression)

eTable 4. Sensitivity Analysis for Incidence and Risk of COVID-19 Severe Disease by Unvaccinated/Partially Vaccinated, Fully Vaccinated and Boosted Populations (Poisson person-days regression)

eTable 5. Waning of Vaccine Effectiveness across the Delta and Omicron Phases (Poisson Person-Days Regressions)

eTable 6. Sensitivity Analysis for Waning of Vaccine Effectiveness Against COVID-19 Severe Disease across the Delta and Omicron Phases (Poisson Person-Days Regressions)

eTable 7. Incidence and Risk of COVID-19 by Unvaccinated/Partially Vaccinated, Fully Vaccinated and Boosted Populations (Calendar-Time Scale Cox Regression)

eTable 8. Incidence and Risk of COVID-19 by Unvaccinated/Partially Vaccinated, Fully Vaccinated and Boosted Populations (Calendar-Time Scale Cox Regression Incorporating Competing Risk)

eTable 9. Waning of Vaccine Effectiveness across the Delta and Omicron Phases (Calendar-Time Scale Cox Regression)

© 2023 Tan WC et al. *JAMA Oncol.*

eTable 10. Waning of Vaccine Effectiveness across the Delta and Omicron Phases (Calendar-Time Scale Cox Regression incorporating Competing Risks)

eTable 11. Regressions with Time Since Last Dose as a Continuous Variable

eTable 12. Person-Days and Incidence Rate/Million Person-Days by Time from Last Vaccine Dose

This supplemental material has been provided by the authors to give readers additional information about their work.

© 2023 Tan WC et al. *JAMA Oncol.*

eFigure 1: Summary timeline of landmark national COVID-19 vaccine recommendations in Singapore

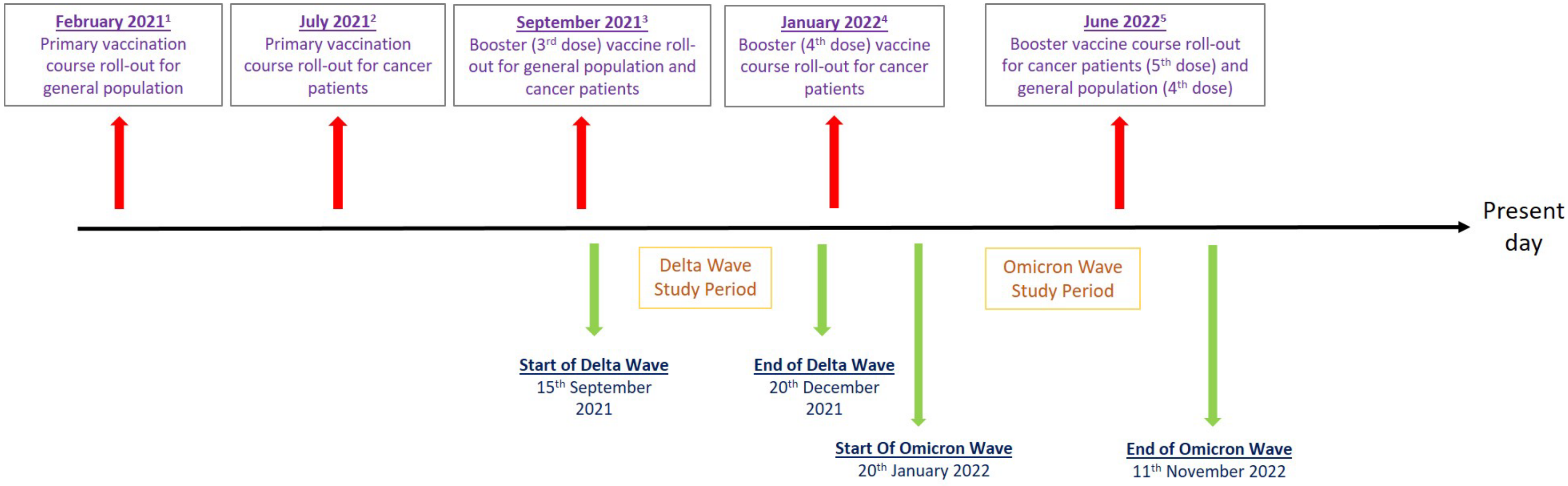

1. Ministry of Health Singapore. Start of COVID-19 Vaccination for Seniors – 19 Feb 2021 (https://www.moh.gov.sg/news-highlights/details/start-of-covid-19-vaccination-for-seniors)
2. Ministry of Health Singapore. Updates to Eligibility for COVID-19 Vaccination – 28th July 2021 (https://www.moh.gov.sg/news-highlights/details/updates-to-eligibility-for-covid-19-vaccination-28jul2021)
3. Ministry of Health Singapore. Expert Committee on COVID-19 Vaccination Recommends an Additional Dose of mRNA COVID-19 Vaccine for Immunocompromised and Seniors – 3rd September 2021 (https://www.moh.gov.sg/news-highlights/details/expert-committee-on-covid-19-vaccination-recommends-an-additional-dose-of-mrna-covid-19-vaccine-for-immunocompromised-and-seniors_3Sep2021)
4. Ministry of Health Singapore. Expert Committee on COVID-19 Vaccination Recommends Booster Vaccination at No Later than Nine Months – 5th January 2022 (https://www.moh.gov.sg/news-highlights/details/expert-committee-on-covid-19-vaccination-recommends-booster-vaccination-at-no-later-than-nine-months)
5. Ministry of Health Singapore. Group Eligible for Second Booster and Dosing Interval for COVID-19 Primary Vaccination Series – 10th June 2022 (https://www.moh.gov.sg/news-highlights/details/groups-eligible-for-second-booster-and-dosing-interval-for-covid-19-primary-vaccination-series)

© 2023 Tan WC et al. *JAMA Oncol.*

eFigure 2: Cumulative incidence rate of COVID-19 infections in actively-treated cancer patients, cancer survivors and matched controls during Delta and Omicron COVID-19 variant infection waves.

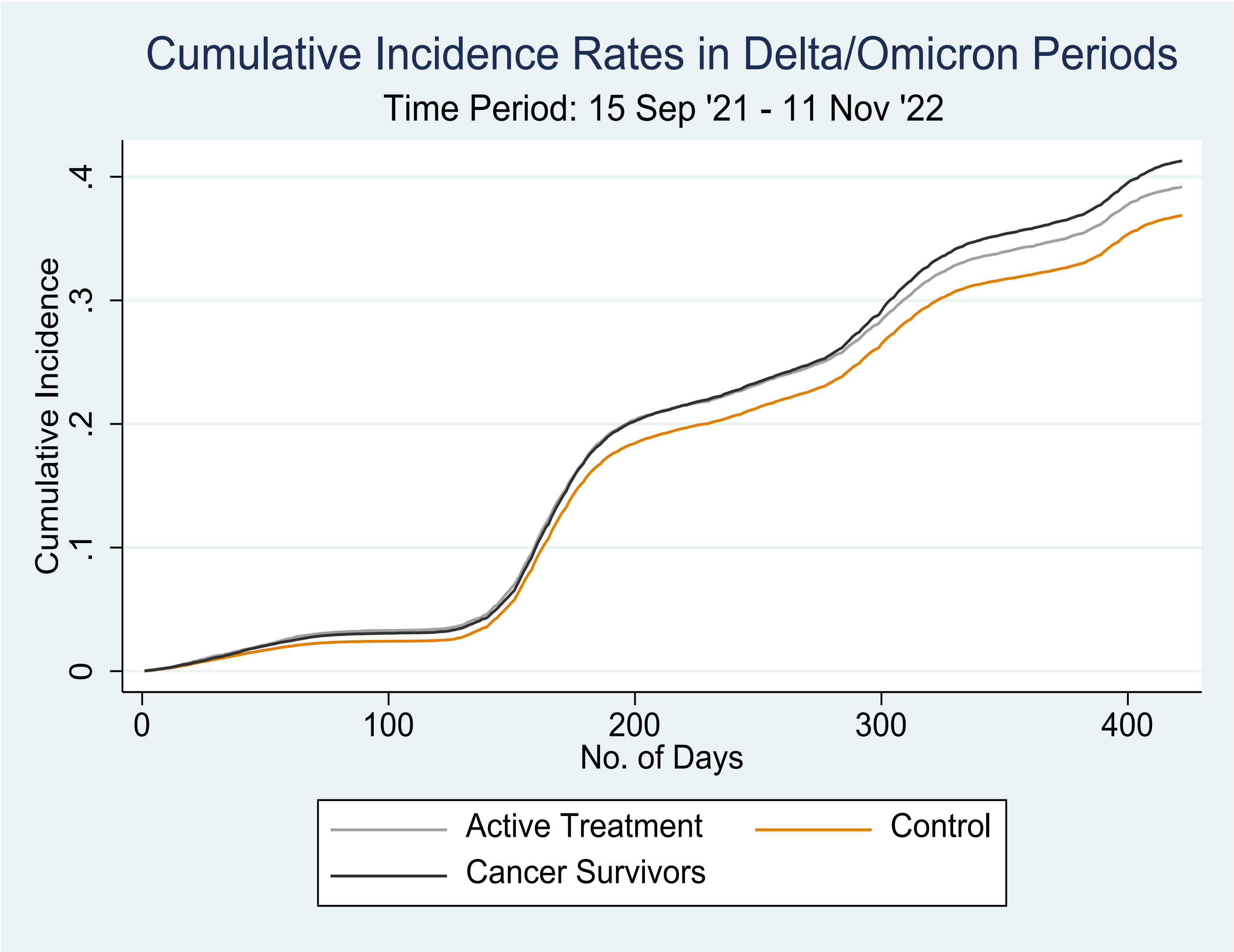

© 2023 Tan WC et al. *JAMA Oncol.*

eTable 1: Person-Days and Incidence Rate per Million Person-Days

Delta Wave												
	**Person-Days**			**COVID-19 Incidence (Incidence Rate/Million Person-Days)**			**COVID-19 Hospitalization (Incidence Rate/Million Person-Days)**			**COVID-19 Severe Disease (Incidence Rate/Million Person-Days)**		
	**Active Treatment**	**Cancer Survivors**	**Control**	**Active Treatment**	**Cancer Survivors**	**Control**	**Active Treatment**	**Cancer Survivors**	**Control**	**Active Treatment**	**Cancer Survivors**	**Control**
**0/1 dose**	174,717	200,518	2,777,029	389	514	203	332	394	134	120	274	84
**2 doses**	1,410,784	2,857,884	21,594,757	495	442	633	179	98	86	29	21	20
**3 doses**	425,185	1,380,236	9,209,298	162	147	145	45	28	13	5	4	2
**Omicron Wave**												
	**Person-Days**			**COVID-19 Incidence (Incidence Rate/Million Person-Days)**			**COVID-19 Hospitalization (Incidence Rate/Million Person-Days)**			**COVID-19 Severe Disease (Incidence Rate/Million Person-Days)**		
	**Active Treatment**	**Cancer Survivors**	**Control**	**Active Treatment**	**Cancer Survivors**	**Control**	**Active Treatment**	**Cancer Survivors**	**Control**	**Active Treatment**	**Cancer Survivors**	**Control**
**0/1 dose**	66,420	128,961	2,233,818	1731	1551	680	-*	-*	-*	196	240	94
**2 doses**	507,842	626,008	6,245,103	2479	2356	2066	697	506	274	100	134	74
**3 doses**	3,308,622	7,991,701	58,212,054	1724	1718	1510	253	115	61	29	21	11
**4 doses**	423,179	1,282,133	8,349,821	1179	1243	1056	151	106	75	21	17	11
*Person-Days for COVID-19 hospitalization not shown, as interpretation is confounded by mandatory inpatient management of unvaccinated/partially-vaccinated patients with confirmed COVID-19 infection												

Note: Table supplementing IRR numbers presented in the main paper.

© 2023 Tan WC et al. *JAMA Oncol.*

eTable 2: Waning of Vaccine Effectiveness across the Delta and Omicron Phases (Competing-Risks Person-Days Regression)

Delta Phase [a]									
	COVID-19 Incidence (95% CI)			COVID-19 Hospitalization (95% CI)			COVID-19 Severe Disease (95% CI)		
	Active Treatment	Cancer Survivors	Control	Active Treatment	Cancer Survivors	Control	Active Treatment	Cancer Survivors	Control
2 doses (8-59 days)	1.00 (.-.)	1.00 (.-.)	1.00 (.-.)	1.00 (.-.)	1.00 (.-.)	1.00 (.-.)	1.00 (.-.)	1.00 (.-.)	1.00 (.-.)
2 doses (60-149 days)	1.43 (1.16-1.76)	1.20 (1.00-1.44)	1.77 (1.65-1.91)	0.89 (0.65-1.22)	0.67 (0.48-0.93)	1.01 (0.86-1.18)	0.77 (0.37-1.59)	0.88 (0.40-1.92)	0.99 (0.72-1.37)
2 doses (>150 days)	1.83 (1.47-2.28)	1.28 (1.06-1.54)	2.87 (2.68-3.08)	1.09 (0.78-1.53)	0.81 (0.58-1.12)	1.71 (1.48-1.98)	0.83 (0.36-1.90)	1.17 (0.54-2.53)	1.76 (1.30-2.38)
3 doses (8-59 days)	0.53 (0.39-0.72)	0.41 (0.33-0.52)	0.54 (0.49-0.59)	0.21 (0.12-0.37)	0.19 (0.13-0.29)	0.17 (0.13-0.21)	0.13 (0.03-0.57)	0.15 (0.05-0.45)	0.09 (0.05-0.15)
3 doses (60-149 days)	0.47 (0.19-1.16)	0.39 (0.22-0.66)	0.75 (0.62-0.91)	0.33 (0.10-1.08)	0.04 (0.01-0.30)	0.17 (0.10-0.32)	0.00 (0.00-0.00)	0.00 (0.00-0.00)	0.11 (0.03-0.46)
**Omicron Phase [b]**									
	COVID-19 Incidence (95% CI)			COVID-19 Hospitalization (95% CI)			COVID-19 Severe Disease (95% CI)		
	Active Treatment	Cancer Survivors	Control	Active Treatment	Cancer Survivors	Control	Active Treatment	Cancer Survivors	Control
2 doses (8-59 days)	1.22 (0.88-1.69)	1.07 (0.76-1.51)	0.97 (0.79-1.18)	1.65 (0.83-3.26)	3.69 (1.99-6.84)	3.74 (2.39-5.85)	8.83 (2.31-33.7)	8.76 (3.46-22.2)	1.98 (0.47-8.30)
2 doses (60-149 days)	1.28 (1.14-1.44)	1.50 (1.34-1.67)	1.47 (1.38-1.56)	2.10 (1.62-2.71)	4.14 (3.08-5.56)	4.21 (3.43-5.18)	5.20 (2.25-12.0)	4.38 (2.37-8.10)	6.77 (4.33-10.59)
2 doses (>150 days)	1.23 (1.11-1.36)	1.48 (1.35-1.61)	1.62 (1.56-1.70)	2.33 (1.87-2.91)	3.22 (2.47-4.22)	3.15 (2.61-3.79)	4.17 (1.89-9.18)	3.80 (2.19-6.61)	4.33 (2.82-6.66)
3 doses (8-59 days)	1.00 (.-.)	1.00 (.-.)	1.00 (.-.)	1.00 (.-.)	1.00 (.-.)	1.00 (.-.)	1.00 (.-.)	1.00 (.-.)	1.00 (.-.)
3 doses (60-149 days)	1.12 (1.04-1.22)	1.31 (1.23-1.40)	1.38 (1.33-1.43)	0.94 (0.76-1.16)	1.00 (0.78-1.28)	1.03 (0.87-1.23)	1.00 (0.44-2.24)	0.88 (0.52-1.48)	1.07 (0.70-1.62)
3 doses (>150 days)	1.28 (1.16-1.40)	1.53 (1.43-1.64)	1.71 (1.64-1.77)	1.01 (0.80-1.28)	0.84 (0.64-1.09)	0.88 (0.73-1.06)	1.61 (0.73-3.54)	0.58 (0.33-1.01)	0.90 (0.58-1.40)
4 doses (8-59 days)	0.91 (0.77-1.07)	1.13 (1.01-1.26)	1.31 (1.23-1.39)	0.37 (0.22-0.61)	0.35 (0.23-0.53)	0.53 (0.41-0.70)	0.53 (0.13-2.11)	0.26 (0.10-0.65)	0.28 (0.13-0.61)
4 doses (60-149 days)	1.26 (1.08-1.48)	1.57 (1.41-1.74)	1.85 (1.75-1.96)	0.69 (0.46-1.03)	0.56 (0.40-0.79)	0.73 (0.58-0.92)	0.76 (0.23-2.53)	0.40 (0.18-0.86)	0.58 (0.33-1.01)

[a] Population of patients within 8-59 days of receiving a 2nd vaccine dose against COVID-19 used as the reference group

© 2023 Tan WC et al. *JAMA Oncol.*

[b] Populations of patients within 8-59 days of receiving a third vaccine dose against COVID-19 used as reference group

© 2023 Tan WC et al. *JAMA Oncol.*

eTable 3: Incidence and Risk of COVID-19 by Unvaccinated/Partially Vaccinated, Fully Vaccinated and Boosted Populations (Poisson person-days regression)

Delta Wave									
	**COVID-19 Incidence (95% CI)**			**COVID-19 Hospitalization (95% CI)**			**COVID-19 Severe Disease (95% CI)**		
**Vaccine doses**	**Active Treatment**	**Cancer Survivors**	**Control**	**Active Treatment**	**Cancer Survivors**	**Controls**	**Active Treatment**	**Cancer Survivors**	**Controls**
**0/1 dose**	0.70 (0.55-0.90)	1.02 (0.84-1.25)	0.28 (0.26-0.31)	1.65 (1.23-2.20)	2.92 (2.26-3.78)	0.99 (0.88-1.10)	3.86 (2.24-6.65)	10.07 (6.87-14.75)	2.51 (2.13-2.96)
**2 doses**	1.00 (.-.)	1.00 (.-.)	1.00 (.-.)	1.00 (.-.)	1.00 (.-.)	1.00 (.-.)	1.00 (.-.)	1.00 (.-.)	1.00 (.-.)
**3 doses**	0.37 (0.29-0.48)	0.34 (0.29-0.40)	0.26 (0.25-0.28)	0.23 (0.15-0.38)	0.23 (0.16-0.33)	0.14 (0.11-0.16)	0.14 (0.03-0.58)	0.13 (0.05-0.32)	0.07 (0.04-0.12)
**Omicron Wave**									
	**COVID-19 Incidence (95% CI)**			**COVID-19 Hospitalization (95% CI)**			**COVID-19 Severe Disease (95% CI)**		
**Vaccine doses**	**Active Treatment**	**Cancer Survivors**	**Control**	**Active Treatment**	**Cancer Survivors**	**Control**	**Active Treatment**	**Cancer Survivors**	**Control**
**0/1 dose**	0.81 (0.66-0.98)	0.77 (0.66-0.89)	0.38 (0.36-0.40)	-*	-*	-*	1.85 (0.99-3.46)	1.56 (1.02-2.38)	0.80 (0.66-0.97)
**2 doses**	1.00 (.-.)	1.00 (.-.)	1.00 (.-.)	1.00 (.-.)	1.00 (.-.)	1.00 (.-.)	1.00 (.-.)	1.00 (.-.)	1.00 (.-.)
**3 doses**	0.84 (0.79-0.90)	0.85 (0.80-0.90)	0.87 (0.85-0.89)	0.43 (0.38-.49)	0.26 (0.23-0.30)	0.28 (0.26-0.30)	0.27 (0.19-0.38)	0.18 (0.13-0.24)	0.18 (0.16-0.21)
**4 doses**	0.72 (0.64-0.81)	0.74 (0.68-0.80)	0.81 (0.79-0.84)	0.23 (0.17-0.31)	0.15 (0.12-0.18)	0.19 (0.18-0.22)	0.12 (0.05-0.28)	0.10 (0.06-0.17)	0.11 (0.09-0.15)
*IRR for COVID-19 hospitalization not shown, as interpretation is confounded by mandatory inpatient management of unvaccinated/partially-vaccinated patients with confirmed COVID-19 infection									

Note: Poisson regressions conducted using methods previously described by Bar-On et al[17].

© 2023 Tan WC et al. *JAMA Oncol.*

eTable 4: Sensitivity Analysis for Incidence and Risk of COVID-19 Severe Disease by Unvaccinated/Partially Vaccinated, Fully Vaccinated and Boosted Populations (Poisson person-days regression)

**Delta Wave**	**COVID-19 Severe Disease (95% CI)**		
**Vaccine doses**	**Active Treatment**	**Cancer Survivors**	**Controls**
**0/1 dose**	4.13 (2.44-6.98)	10.55 (7.27-15.32)	2.54 (2.16-2.98)
**2 doses**	1.00 (.-.)	1.00 (.-.)	1.00 (.-.)
**3 doses**	0.13 (0.03-0.56)	0.15 (0.07-0.35)	0.07 (0.04-0.11)
**Omicron Wave**	**COVID-19 Severe Disease (95% CI)**		
**Vaccine doses**	**Active Treatment**	**Cancer Survivors**	**Control**
**0/1 dose**	2.13 (1.20-3.79)	1.53 (1.02-2.28)	0.82 (0.69-0.99)
**2 doses**	1.00 (.-.)	1.00 (.-.)	1.00 (.-.)
**3 doses**	0.28 (0.20-0.39)	0.17 (0.13-0.22)	0.19 (0.17-0.22)
**4 doses**	0.11 (0.05-0.26)	0.09 (0.06-0.15)	0.11 (0.09-0.14)

Note: Supplementary sensitivity analysis on severity outcomes. Poisson regressions conducted using methods previously described by Bar-On et al[17], taking into consideration the competing risk for those who died from other causes (presumably cancer) after they have been infected with COVID.

© 2023 Tan WC et al. *JAMA Oncol.*

eTable 5: Waning of Vaccine Effectiveness across the Delta and Omicron Phases (Poisson Person-Days Regressions)

Delta Phase [a]									
	COVID-19 Incidence (95% CI)			COVID-19 Hospitalization (95% CI)			COVID-19 Severe Disease (95% CI)		
	Active Treatment	Cancer Survivors	Control	Active Treatment	Cancer Survivors	Control	Active Treatment	Cancer Survivors	Control
2 doses (8-59 days)	1.00 (.-.)	1.00 (.-.)	1.00 (.-.)	1.00 (.-.)	1.00 (.-.)	1.00 (.-.)	1.00 (.-.)	1.00 (.-.)	1.00 (.-.)
2 doses (60-149 days)	1.43 (1.16-1.76)	1.20 (1.00-1.44)	1.77 (1.65-1.91)	0.89 (0.65-1.22)	0.67 (0.48-0.94)	1.01 (0.87-1.18)	0.76 (0.37-1.57)	0.87 (0.40-1.91)	0.98 (0.71-1.34)
2 doses (>150 days)	1.83 (1.47-2.28)	1.28 (1.06-1.54)	2.89 (2.69-3.11)	1.10 (0.78-1.54)	0.81 (0.58-1.13)	1.74 (1.51-2.01)	0.83 (0.36-1.90)	1.23 (0.57-2.64)	1.78 (1.32-2.40)
3 doses (8-59 days)	0.53 (0.39-0.72)	0.41 (0.33-0.52)	0.53 (0.49-0.58)	0.21 (0.12-0.37)	0.19 (0.13-0.29)	0.17 (0.13-0.21)	0.12 (0.03-0.56)	0.15 (0.05-0.45)	0.09 (0.05-0.15)
3 doses (60-149 days)	0.46 (0.19-1.15)	0.38 (0.22-0.66)	0.75 (0.62-0.91)	0.33 (0.10-1.07)	0.04 (0.01-0.30)	0.18 (0.10-0.33)	0.00 (0.00-0.00)	0.00 (0.00-0.00)	0.10 (0.02-0.43)
Omicron Phase [b]									
	COVID-19 Incidence (95% CI)			COVID-19 Hospitalization (95% CI)			COVID-19 Severe Disease (95% CI)		
	Active Treatment	Cancer Survivors	Control	Active Treatment	Cancer Survivors	Control	Active Treatment	Cancer Survivors	Control
2 doses (8-59 days)	1.34 (0.97-1.86)	1.21 (0.85-1.71)	1.11 (0.97-1.28)	1.87 (0.95-3.69)	4.16 (2.25-7.69)	4.30 (3.19-5.81)	10.00 (2.63-38.05)	10.19 (4.02-25.81)	3.65 (1.56-8.50)
2 doses (60-149 days)	1.43 (1.27-1.61)	1.64 (1.46-1.83)	1.58 (1.51-1.65)	2.22 (1.72-2.87)	4.40 (3.28-5.89)	4.48 (3.88-5.18)	5.50 (2.38-12.69)	4.95 (2.70-9.07)	8.93 (6.43-12.40)
2 doses (>150 days)	1.38 (1.25-1.53)	1.59 (1.46-1.74)	1.77 (1.71-1.82)	2.55 (2.04-3.18)	3.52 (2.69-4.60)	3.17 (2.79-3.62)	4.89 (2.23-10.71)	4.36 (2.52-7.55)	5.41 (3.93-7.44)
3 doses (8-59 days)	1.00 (.-.)	1.00 (.-.)	1.00 (.-.)	1.00 (.-.)	1.00 (.-.)	1.00 (.-.)	1.00 (.-.)	1.00 (.-.)	1.00 (.-.)
3 doses (60-149 days)	1.15 (1.06-1.25)	1.32 (1.24-1.41)	1.43 (1.40-1.47)	0.96 (0.78-1.18)	1.03 (0.81-1.31)	1.00 (0.88-1.13)	1.07 (0.48-2.39)	0.92 (0.55-1.55)	1.18 (0.86-1.61)
3 doses (>150 days)	1.31 (1.20-1.44)	1.55 (1.44-1.67)	1.80 (1.75-1.85)	1.05 (0.83-1.32)	0.88 (0.67-1.14)	0.86 (0.75-0.97)	1.70 (0.78-3.72)	0.61 (0.35-1.06)	1.03 (0.75-1.43)
4 doses (8-59 days)	0.92 (0.78-1.09)	1.13 (1.01-1.26)	1.37 (1.31-1.43)	0.38 (0.23-0.63)	0.36 (0.24-0.55)	0.47 (0.38-0.57)	0.55 (0.14-2.20)	0.27 (0.11-0.68)	0.46 (0.27-0.77)
4 doses (60-149 days)	1.29 (1.10-1.50)	1.56 (1.40-1.73)	1.98 (1.90-2.06)	0.71 (0.47-1.07)	0.59 (0.41-0.83)	0.71 (0.61-0.84)	0.80 (0.24-2.66)	0.45 (0.21-0.97)	0.76 (0.50-1.15)
[a] Population of patients within 8-59 days of receiving a 2nd vaccine dose against COVID-19 used as the reference group									

© 2023 Tan WC et al. *JAMA Oncol.*

[b] Populations of patients within 8-59 days of receiving a third vaccine dose against COVID-19 used as reference group

Note: Poisson regressions conducted using methods previously described by Bar-On et al[17], looking at waning.

© 2023 Tan WC et al. *JAMA Oncol.*

eTable 6: Sensitivity Analysis for Waning of Vaccine Effectiveness Against COVID-19 Severe Disease across the Delta and Omicron Phases (Poisson Person-Days Regressions)

	COVID-19 Severe Disease (95% CI)		
	**Active Treatment**	**Cancer Survivors**	**Control**
2 doses (8-59 days)	1.00 (.-.)	1.00 (.-.)	1.00 (.-.)
2 doses (60-149 days)	0.80 (0.39-1.63)	0.88 (0.40-1.92)	1.01 (0.73-1.38)
2 doses (>150 days)	0.82 (0.36-1.88)	1.28 (0.60-2.74)	1.81 (1.35-2.44)
3 doses (8-59 days)	0.12 (0.03-0.55)	0.18 (0.06-0.51)	0.09 (0.05-0.15)
3 doses (60-149 days)	0.00 (0.00-0.00)	0.00 (0.00-0.00)	0.10 (0.03-0.44)
	**COVID-19 Severe Disease (95% CI)**		
	**Active Treatment**	**Cancer Survivors**	**Control**
2 doses (8-59 days)	8.42 (2.31-30.76)	3.95 (0.90-17.39)	4.70 (2.23-9.88)
2 doses (60-149 days)	4.79 (2.23-10.31)	13.60 (7.62-24.30)	8.96 (6.49-12.37)
2 doses (>150 days)	4.40 (2.16-8.98)	9.13 (5.19-16.05)	5.63 (4.12-7.68)
3 doses (8-59 days)	1.00 (.-.)	1.00 (.-.)	1.00 (.-.)
3 doses (60-149 days)	0.95 (0.46-1.98)	2.04 (1.16-3.57)	1.30 (0.95-1.76)
3 doses (>150 days)	1.56 (0.77-3.16)	1.52 (0.86-2.71)	1.08 (0.79-1.48)
4 doses (8-59 days)	0.46 (0.12-1.77)	0.54 (0.21-1.39)	0.46 (0.28-0.77)
4 doses (60-149 days)	0.63 (0.20-2.03)	1.62 (0.82-3.20)	0.74 (0.50-1.11)

Note: Supplementary sensitivity analysis on severity outcomes. Poisson regressions conducted using methods previously described by Bar-On et al[17], looking into waning and taking into consideration the competing risk for those who died from other causes (presumably cancer) after they have been infected with COVID.

© 2023 Tan WC et al. *JAMA Oncol.*

eTable 7: Incidence and Risk of COVID-19 by Unvaccinated/Partially Vaccinated, Fully Vaccinated and Boosted Populations (Calendar-Time Scale Cox Regression)

Delta Wave									
	COVID-19 Incidence (95% CI)			COVID-19 Hospitalization (95% CI)			COVID-19 Severe Disease (95% CI)		
Vaccine doses	Active Treatment	Cancer Survivors	Controls	Active Treatment	Cancer Survivors	Controls	Active Treatment	Cancer Survivors	Controls
0/1 dose	0.69 (0.53-0.91)	1.06 (0.86-1.31)	0.27 (0.25-0.30)	1.68 (1.22-2.30)	3.51 (2.69-4.58)	1.22 (1.09-1.37)	4.49 (2.59-7.79)	11.60 (7.92-16.99)	3.21 (2.72-3.78)
2 doses	1.00 (.-.)	1.00 (.-.)	1.00 (.-.)	1.00 (.-.)	1.00 (.-.)	1.00 (.-.)	1.00 (.-.)	1.00 (.-.)	1.00 (.-.)
3 doses	0.37 (0.29-0.48)	0.35 (0.30-0.41)	0.26 (0.25-0.28)	0.26 (0.16-0.42)	0.26 (0.18-0.38)	0.17 (0.14-0.21)	0.17 (0.04-0.69)	0.15 (0.06-0.39)	0.10 (0.06-0.16)
Omicron Wave									
	COVID-19 Incidence (95% CI)			COVID-19 Hospitalization (95% CI)			COVID-19 Severe Disease (95% CI)		
Vaccine doses	Active Treatment	Cancer Survivors	Control	Active Treatment	Cancer Survivors	Control	Active Treatment	Cancer Survivors	Control
0/1 dose	0.75 (0.62-0.92)	0.69 (0.60-0.80)	0.38 (0.36-0.40)	-*	-*	-*	1.96 (1.06-3.66)	1.66 (1.09-2.55)	1.18 (1.00-1.40)
2 doses	1.00 (.-.)	1.00 (.-.)	1.00 (.-.)	1.00 (.-.)	1.00 (.-.)	1.00 (.-.)	1.00 (.-.)	1.00 (.-.)	1.00 (.-.)
3 doses	0.80 (0.75-0.86)	0.79 (0.74-0.83)	0.82 (0.80-0.83)	0.42 (0.36-0.48)	0.24 (0.21-0.28)	0.25 (0.23-0.26)	0.27 (0.18-0.39)	0.17 (0.12-0.22)	0.16 (0.14-0.19)
4 doses	0.66 (0.58-0.74)	0.65 (0.60-0.71)	0.72 (0.70-0.75)	0.26 (0.19-0.35)	0.21 (0.17-0.27)	0.31 (0.28-0.34)	0.16 (0.07-0.35)	0.15 (0.09-0.25)	0.18 (0.14-0.23)
*IRR for COVID-19 hospitalization not shown, as interpretation is confounded by mandatory inpatient management of unvaccinated/partially-vaccinated patients with confirmed COVID-19 infection									

Note: Calendar time-scale cox regressions conducted with reference to Lund et al[35].

© 2023 Tan WC et al. *JAMA Oncol.*

eTable 8: Incidence and Risk of COVID-19 by Unvaccinated/Partially Vaccinated, Fully Vaccinated and Boosted Populations (Calendar-Time Scale Cox Regression Incorporating Competing Risk)

Delta Wave									
	COVID-19 Incidence (95% CI)			COVID-19 Hospitalization (95% CI)			COVID-19 Severe Disease (95% CI)		
Vaccine doses	Active Treatment	Cancer Survivors	Controls	Active Treatment	Cancer Survivors	Controls	Active Treatment	Cancer Survivors	Controls
0/1 dose	0.64 (0.49-0.85)	1.01 (0.82-1.26)	0.27 (0.25-0.29)	1.54 (1.12-2.11)	3.34 (2.56-4.36)	1.22 (1.08-1.37)	4.07 (2.32-7.15)	11.00 (7.48-16.17)	3.21 (2.72-3.78)
2 doses	1.00 (.-.)	1.00 (.-.)	1.00 (.-.)	1.00 (.-.)	1.00 (.-.)	1.00 (.-.)	1.00 (.-.)	1.00 (.-.)	1.00 (.-.)
3 doses	0.38 (0.29-0.49)	0.35 (0.30-0.41)	0.26 (0.25-0.28)	0.27 (0.17-0.43)	0.27 (0.19-0.38)	0.17 (0.14-0.21)	0.17 (0.04-0.73)	0.16 (0.06-0.39)	0.10 (0.06-0.16)
**Omicron Wave**									
	COVID-19 Incidence (95% CI)			COVID-19 Hospitalization (95% CI)			COVID-19 Severe Disease (95% CI)		
Vaccine doses	Active Treatment	Cancer Survivors	Control	Active Treatment	Cancer Survivors	Control	Active Treatment	Cancer Survivors	Control
0/1 dose	0.71 (0.59-0.87)	0.67 (0.58-0.79)	0.38 (0.36-0.40)	-*	-*	-*	1.64 (1.07-2.50)	1.90 (1.01-3.57)	1.18 (1.00-1.40)
2 doses	1.00 (.-.)	1.00 (.-.)	1.00 (.-.)	1.00 (.-.)	1.00 (.-.)	1.00 (.-.)	1.00 (.-.)	1.00 (.-.)	1.00 (.-.)
3 doses	0.87 (0.82-0.93)	0.84 (0.79-0.89)	0.84 (0.82-0.85)	0.46 (0.40-0.52)	0.26 (0.23-0.30)	0.26 (0.24-0.27)	0.18 (0.14-0.24)	0.30 (0.22-0.43)	0.16 (0.14-0.19)
4 doses	0.77 (0.68-0.86)	0.72 (0.66-0.78)	0.75 (0.72-0.77)	0.31 (0.23-0.41)	0.24 (0.19-0.30)	0.32 (0.29-0.35)	0.17 (0.10-0.28)	0.19 (0.09-0.44)	0.18 (0.14-0.23)
*IRR for COVID-19 hospitalization not shown, as interpretation is confounded by mandatory inpatient management of unvaccinated/partially-vaccinated patients with confirmed COVID-19 infection									

Note: Calendar time-scale cox regressions conducted with reference to Lund et al[35], taking into consideration competing risks.

eTable 9: Waning of Vaccine Effectiveness across the Delta and Omicron Phases (Calendar-Time Scale Cox Regression)

**Delta Phase [a]**									
	**COVID-19 Incidence (95% CI)**			**COVID-19 Hospitalization (95% CI)**			**COVID-19 Severe Disease (95% CI)**		
	**Active Treatment**	**Cancer Survivors**	**Control**	**Active Treatment**	**Cancer Survivors**	**Control**	**Active Treatment**	**Cancer Survivors**	**Control**
2 doses (8-59 days)	1.00 (.-.)	1.00 (.-.)	1.00 (.-.)	1.00 (.-.)	1.00 (.-.)	1.00 (.-.)	1.00 (.-.)	1.00 (.-.)	1.00 (.-.)
2 doses (60-149 days)	1.41 (1.14-1.74)	1.17 (0.97-1.40)	1.77 (1.64-1.90)	0.85 (0.62-1.16)	0.60 (0.43-0.83)	0.81 (0.69-0.95)	0.73 (0.34-1.56)	0.79 (0.36-1.74)	0.76 (0.55-1.05)
2 doses (>150 days)	1.85 (1.49-2.31)	1.30 (1.07-1.57)	3.04 (2.83-3.27)	1.13 (0.81-1.59)	0.77 (0.55-1.08)	1.68 (1.45-1.94)	0.90 (0.39-2.08)	1.22 (0.57-2.62)	1.64 (1.21-2.22)
3 doses (8-59 days)	0.55 (0.40-0.75)	0.42 (0.34-0.53)	0.56 (0.51-0.61)	0.24 (0.14-0.42)	0.20 (0.13-0.32)	0.20 (0.16-0.26)	0.16 (0.03-0.71)	0.18 (0.06-0.55)	0.11 (0.06-0.19)
3 doses (60-149 days)	0.45 (0.16-1.24)	0.37 (0.20-0.67)	0.81 (0.66-1.00)	0.29 (0.07-1.25)	0.00 (0.00-0.00)	0.27 (0.15-0.50)	0.00 (0.00-0.00)	0.00 (0.00-0.00)	0.13 (0.03-0.56)
**Omicron Phase [b]**									
	**COVID-19 Incidence (95% CI)**			**COVID-19 Hospitalization (95% CI)**			**COVID-19 Severe Disease (95% CI)**		
	**Active Treatment**	**Cancer Survivors**	**Control**	**Active Treatment**	**Cancer Survivors**	**Control**	**Active Treatment**	**Cancer Survivors**	**Control**
2 doses (8-59 days)	1.38 (1.00-1.91)	1.23 (0.88-1.73)	1.06 (0.93-1.21)	1.85 (0.94-3.63)	4.94 (2.69-9.07)	4.89 (3.68-6.50)	9.18 (2.47-34.10)	12.39 (4.83-31.78)	5.89 (3.01-11.51)
2 doses (60-149 days)	1.42 (1.26-1.60)	1.62 (1.45-1.81)	1.53 (1.47-1.60)	2.24 (1.74-2.90)	5.62 (4.20-7.51)	5.46 (4.77-6.24)	4.97 (2.18-11.33)	6.29 (3.33-11.87)	10.57 (7.83-14.28)
2 doses (>150 days)	1.39 (1.26-1.54)	1.57 (1.44-1.72)	1.73 (1.68-1.79)	2.52 (2.02-3.15)	4.13 (3.16-5.40)	3.63 (3.22-4.09)	4.31 (2.02-9.19)	5.51 (3.11-9.75)	6.07 (4.57-8.05)
3 doses (8-59 days)	1.00 (.-.)	1.00 (.-.)	1.00 (.-.)	1.00 (.-.)	1.00 (.-.)	1.00 (.-.)	1.00 (.-.)	1.00 (.-.)	1.00 (.-.)
3 doses (60-149 days)	1.13 (1.05-1.23)	1.27 (1.19-1.35)	1.37 (1.34-1.40)	0.96 (0.78-1.19)	1.23 (0.96-1.57)	1.09 (0.97-1.22)	0.95 (0.44-2.07)	1.22 (0.71-2.10)	1.27 (0.96-1.69)
3 doses (>150 days)	1.28 (1.17-1.41)	1.40 (1.31-1.51)	1.62 (1.58-1.67)	1.06 (0.84-1.34)	1.08 (0.83-1.40)	0.96 (0.84-1.08)	1.52 (0.70-3.31)	0.81 (0.45-1.47)	1.07 (0.79-1.44)
4 doses (8-59 days)	0.85 (0.72-1.00)	0.96 (0.86-1.07)	1.15 (1.10-1.20)	0.40 (0.24-0.67)	0.58 (0.38-0.88)	0.77 (0.63-0.93)	0.58 (0.15-2.30)	0.47 (0.18-1.20)	0.60 (0.36-1.00)
4 doses (60-149 days)	1.19 (1.02-1.40)	1.30 (1.17-1.44)	1.62 (1.55-1.69)	0.86 (0.57-1.29)	1.06 (0.74-1.53)	1.36 (1.15-1.61)	0.65 (0.16-2.61)	1.09 (0.48-2.48)	1.38 (0.91-2.08)
[a] Population of patients within 8-59 days of receiving a 2nd vaccine dose against COVID-19 used as the reference group									

© 2023 Tan WC et al. *JAMA Oncol.*

[b] Populations of patients within 8-59 days of receiving a third vaccine dose against COVID-19 used as reference group

Note: Calendar time-scale cox regressions conducted with reference to Lund et al[35], looking at waning.

eTable 10: Waning of Vaccine Effectiveness across the Delta and Omicron Phases (Calendar-Time Scale Cox Regression incorporating Competing Risks)

	Delta Phase [a]								
	COVID-19 Incidence (95% CI)			COVID-19 Hospitalization (95% CI)			COVID-19 Severe Disease (95% CI)		
	Active Treatment	Cancer Survivors	Control	Active Treatment	Cancer Survivors	Control	Active Treatment	Cancer Survivors	Control
2 doses (8-59 days)	1.00 (.-.)	1.00 (.-.)	1.00 (.-.)	1.00 (.-.)	1.00 (.-.)	1.00 (.-.)	1.00 (.-.)	1.00 (.-.)	1.00 (.-.)
2 doses (60-149 days)	1.42 (1.15-1.75)	1.17 (0.97-1.41)	1.77 (1.65-1.90)	0.85 (0.62-1.17)	0.60 (0.43-0.83)	0.81 (0.69-0.95)	0.74 (0.35-1.55)	0.80 (0.36-1.76)	0.76 (0.55-1.06)
2 doses (>150 days)	1.86 (1.49-2.32)	1.30 (1.07-1.57)	3.04 (2.83-3.27)	1.14 (0.81-1.59)	0.77 (0.55-1.08)	1.68 (1.44-1.95)	0.90 (0.39-2.08)	1.22 (0.56-2.65)	1.64 (1.21-2.23)
3 doses (8-59 days)	0.55 (0.41-0.76)	0.43 (0.34-0.53)	0.56 (0.51-0.61)	0.24 (0.14-0.43)	0.21 (0.13-0.32)	0.20 (0.16-0.26)	0.16 (0.03-0.73)	0.18 (0.06-0.56)	0.11 (0.06-0.19)
3 doses (60-149 days)	0.45 (0.16-1.26)	0.37 (0.20-0.68)	0.82 (0.67-1.00)	0.30 (0.07-1.27)	0.00 (0.00-0.00)	0.27 (0.15-0.50)	0.00 (0.00-0.00)	0.00 (0.00-0.00)	0.13 (0.03-0.56)
	Omicron Phase [b]								
	COVID-19 Incidence (95% CI)			COVID-19 Hospitalization (95% CI)			COVID-19 Severe Disease (95% CI)		
	Active Treatment	Cancer Survivors	Control	Active Treatment	Cancer Survivors	Control	Active Treatment	Cancer Survivors	Control
2 doses (8-59 days)	1.36 (0.99-1.87)	1.21 (0.87-1.70)	1.05 (0.92-1.20)	1.82 (0.92-3.58)	4.85 (2.63-8.95)	4.81 (3.63-6.39)	8.91 (2.40-33.04)	12.19 (4.75-31.28)	5.78 (2.96-11.31)
2 doses (60-149 days)	1.38 (1.23-1.55)	1.58 (1.42-1.77)	1.52 (1.45-1.58)	2.18 (1.69-2.81)	5.48 (4.10-7.32)	5.38 (4.71-6.16)	4.78 (2.14-10.68)	6.12 (3.26-11.49)	10.42 (7.72-14.07)
2 doses (>150 days)	1.34 (1.21-1.49)	1.53 (1.40-1.67)	1.72 (1.67-1.78)	2.44 (1.95-3.04)	4.01 (3.07-5.25)	3.61 (3.19-4.07)	4.15 (1.96-8.79)	5.35 (3.01-9.48)	6.03 (4.50-8.07)
3 doses (8-59 days)	1.00 (.-.)	1.00 (.-.)	1.00 (.-.)	1.00 (.-.)	1.00 (.-.)	1.00 (.-.)	1.00 (.-.)	1.00 (.-.)	1.00 (.-.)
3 doses (60-149 days)	1.14 (1.05-1.23)	1.27 (1.19-1.35)	1.37 (1.34-1.40)	0.97 (0.78-1.19)	1.23 (0.96-1.57)	1.09 (0.97-1.22)	0.96 (0.44-2.07)	1.22 (0.71-2.10)	1.28 (0.96-1.70)
3 doses (>150 days)	1.32 (1.21-1.45)	1.43 (1.34-1.54)	1.63 (1.59-1.68)	1.11 (0.88-1.41)	1.12 (0.85-1.47)	0.97 (0.85-1.10)	1.60 (0.74-3.47)	0.85 (0.46-1.55)	1.08 (0.79-1.47)
4 doses (8-59 days)	0.90 (0.76-1.06)	0.99 (0.89-1.10)	1.16 (1.11-1.21)	0.43 (0.25-0.72)	0.61 (0.40-0.93)	0.78 (0.64-0.94)	0.64 (0.16-2.54)	0.50 (0.19-1.29)	0.61 (0.37-1.03)
4 doses (60-149 days)	1.27 (1.09-1.49)	1.34 (1.21-1.49)	1.64 (1.57-1.70)	0.92 (0.60-1.41)	1.13 (0.78-1.64)	1.38 (1.16-1.64)	0.71 (0.17-3.00)	1.17 (0.51-2.66)	1.40 (0.92-2.14)

[a] Population of patients within 8-59 days of receiving a 2nd vaccine dose against COVID-19 used as the reference group

© 2023 Tan WC et al. *JAMA Oncol.*

[b] Populations of patients within 8-59 days of receiving a third vaccine dose against COVID-19 used as reference group

Note: Calendar time-scale cox regressions conducted with reference to Lund et al[35], taking into consideration competing risks, looking at waning.

© 2023 Tan WC et al. *JAMA Oncol.*

eTable 11: Regressions with Time Since Last Dose as a Continuous Variable

Delta Wave									
	**COVID-19 Incidence (95% CI)**			**COVID-19 Hospitalization (95% CI)**			**COVID-19 Severe Disease (95% CI)**		
**Group**	**Active Treatment**	**Cancer Survivors**	**Controls**	**Active Treatment**	**Cancer Survivors**	**Controls**	**Active Treatment**	**Cancer Survivors**	**Controls**
**0/1 dose**	2.45e-05 (-1.08e-04,1.57e-04)	1.14e-04 (-5.72e-06,2.35e-04)	-5.04e-05 (-8.10e-05,-1.98e-05)	1.10e-04 (2.14e-06,2.18e-04)	2.53e-04 (1.60e-04,3.45e-04)	4.61e-05 (2.87e-05,6.35e-05)	6.93e-05 (8.47e-06,1.30e-04)	2.48e-04 (1.74e-04,3.22e-04)	6.32e-05 (5.14e-05,7.50e-05)
**2 doses**	0.00e+00 (.,.)	0.00e+00 (.,.)	0.00e+00 (.,.)	0.00e+00 (.,.)	0.00e+00 (.,.)	0.00e+00 (.,.)	0.00e+00 (.,.)	0.00e+00 (.,.)	0.00e+00 (.,.)
**3 doses**	-2.32e-04 (-4.13e-04,-4.99e-05)	-2.18e-04 (-3.33e-04,-1.03e-04)	-1.39e-04 (-1.80e-04,-9.69e-05)	-1.66e-04 (-2.65e-04,-6.64e-05)	-4.46e-05 (-9.99e-05,1.08e-05)	-2.73e-05 (-4.16e-05,-1.30e-05)	-3.40e-05 (-6.22e-05,-5.73e-06)	-2.45e-05 (-4.28e-05,-6.22e-06)	-1.03e-05 (-1.61e-05,-4.52e-06)
**Days 2 doses**	1.90e-06 (-8.86e-08,3.88e-06)	7.75e-07 (-5.00e-07,2.05e-06)	3.90e-06 (3.39e-06,4.41e-06)	-9.93e-07 (-2.32e-06,3.40e-07)	-3.03e-07 (-9.01e-07,2.94e-07)	7.94e-09 (-1.91e-07,2.06e-07)	-5.11e-07 (-1.15e-06,1.25e-07)	-4.44e-08 (-2.89e-07,2.00e-07)	5.31e-08 (-3.90e-08,1.45e-07)
**Days 3 doses**	4.64e-06 (-3.68e-06,1.30e-05)	-1.48e-06 (-6.28e-06,3.31e-06)	4.04e-06 (2.26e-06,5.82e-06)	-1.14e-06 (-5.32e-06,3.04e-06)	-3.26e-06 (-5.59e-06,-9.27e-07)	-1.11e-06 (-1.69e-06,-5.31e-07)	-1.16e-06 (-2.20e-06,-1.33e-07)	-1.56e-07 (-9.36e-07,6.24e-07)	-2.63e-07 (-5.08e-07,-1.82e-08)
**Days 2 doses squared**	-8.92e-10 (-9.97e-09,8.18e-09)	-3.81e-10 (-5.94e-09,5.17e-09)	-1.72e-09 (-3.99e-09,5.58e-10)	5.43e-09 (-7.53e-10,1.16e-08)	1.45e-09 (-1.15e-09,4.06e-09)	2.00e-09 (1.10e-09,2.90e-09)	2.17e-09 (-7.50e-10,5.10e-09)	4.21e-10 (-6.65e-10,1.51e-09)	2.96e-10 (-1.27e-10,7.19e-10)
**Days 3 doses squared**	-1.92e-08 (-1.05e-07,6.67e-08)	3.48e-08 (-1.36e-08,8.33e-08)	2.69e-09 (-1.56e-08,2.09e-08)	1.87e-08 (-2.58e-08,6.31e-08)	3.15e-08 (8.77e-09,5.43e-08)	1.26e-08 (6.72e-09,1.85e-08)	1.25e-08 (6.63e-10,2.43e-08)	2.31e-09 (-5.59e-09,1.02e-08)	3.53e-09 (9.25e-10,6.14e-09)
**Omicron Wave**									
	**COVID-19 Incidence (95% CI)**			**COVID-19 Hospitalization (95% CI)**			**COVID-19 Severe Disease (95% CI)**		
**Vaccine doses**	**Active Treatment**	**Cancer Survivors**	**Control**	**Active Treatment**	**Cancer Survivors**	**Control**	**Active Treatment**	**Cancer Survivors**	**Control**
**0/1 dose**	-1.24e-04 (-6.30e-04,3.82e-04)	2.04e-04 (-1.79e-04,5.87e-04)	-2.30e-04 (-3.38e-04,-1.22e-04)	-*	-*	-*	1.05e-04 (-2.92e-05,2.40e-04)	1.20e-04 (5.66e-06,2.34e-04)	6.47e-06 (-1.36e-05,2.65e-05)
**2 doses**	0.00e+00 (.,.)	0.00e+00 (.,.)	0.00e+00 (.,.)	0.00e+00 (.,.)	0.00e+00 (.,.)	0.00e+00 (.,.)	0.00e+00 (.,.)	0.00e+00 (.,.)	0.00e+00 (.,.)
**3 doses**	-5.38e-04 (-9.46e-04,-1.30e-04)	-4.04e-04 (-7.26e-04,-8.16e-05)	-3.10e-04 (-4.16e-04,-2.05e-04)	-1.96e-04 (-3.85e-04,-7.50e-06)	-3.42e-04 (-4.82e-04,-2.03e-04)	-1.80e-04 (-2.13e-04,-1.47e-04)	-7.92e-05 (-1.55e-04,-3.55e-06)	-9.22e-05 (-1.64e-04,-2.07e-05)	-4.91e-05 (-6.47e-05,-3.35e-05)

© 2023 Tan WC et al. *JAMA Oncol.*

**4 doses**	-4.79e-04 (-9.85e-04,2.72e-05)	-1.05e-04 (-4.74e-04,2.63e-04)	1.73e-04 (5.04e-05,2.96e-04)	-3.15e-04 (-5.32e-04,-9.83e-05)	-4.70e-04 (-6.19e-04,-3.22e-04)	-2.47e-04 (-2.84e-04,-2.10e-04)	-7.56e-05 (-1.65e-04,1.36e-05)	-1.36e-04 (-2.11e-04,-6.10e-05)	-6.42e-05 (-8.14e-05,-4.70e-05)
**Days 2 doses**	4.39e-06 (8.24e-07,7.95e-06)	8.33e-06 (5.37e-06,1.13e-05)	8.19e-06 (7.31e-06,9.07e-06)	2.57e-06 (8.11e-07,4.32e-06)	6.19e-07 (-7.26e-07,1.96e-06)	3.78e-07 (9.52e-08,6.62e-07)	-2.06e-07 (-9.80e-07,5.69e-07)	4.12e-07 (-2.05e-07,1.03e-06)	1.92e-07 (6.34e-08,3.20e-07)
**Days 3 doses**	5.02e-06 (3.08e-06,6.97e-06)	8.24e-06 (6.96e-06,9.52e-06)	8.32e-06 (7.89e-06,8.76e-06)	6.16e-07 (-1.81e-07,1.41e-06)	4.25e-07 (7.32e-08,7.76e-07)	2.21e-07 (1.25e-07,3.16e-07)	6.79e-08 (-2.09e-07,3.45e-07)	1.72e-08 (-1.34e-07,1.68e-07)	8.22e-08 (4.23e-08,1.22e-07)
**Days 4 doses**	6.68e-06 (-1.65e-06,1.50e-05)	7.14e-06 (2.03e-06,1.22e-05)	9.53e-06 (7.71e-06,1.14e-05)	1.21e-06 (-1.98e-06,4.41e-06)	-6.34e-08 (-1.64e-06,1.51e-06)	3.49e-08 (-5.09e-07,5.79e-07)	-4.58e-07 (-1.82e-06,9.08e-07)	5.79e-08 (-5.96e-07,7.12e-07)	1.33e-07 (-4.95e-08,3.16e-07)
**Days 2 doses squared**	-1.17e-08 (-1.93e-08,-4.18e-09)	-2.03e-08 (-2.68e-08,-1.39e-08)	-1.74e-08 (-1.92e-08,-1.57e-08)	-5.18e-09 (-9.11e-09,-1.25e-09)	-1.77e-09 (-5.07e-09,1.53e-09)	-1.46e-09 (-2.10e-09,-8.29e-10)	9.98e-10 (-1.03e-09,3.03e-09)	-1.41e-09 (-2.63e-09,-1.90e-10)	-6.21e-10 (-8.90e-10,-3.51e-10)
**Days 3 doses squared**	-9.18e-09 (-1.44e-08,-3.91e-09)	-1.72e-08 (-2.06e-08,-1.38e-08)	-1.52e-08 (-1.63e-08,-1.40e-08)	-1.18e-09 (-3.38e-09,1.01e-09)	-1.81e-09 (-2.80e-09,-8.14e-10)	-9.66e-10 (-1.23e-09,-7.07e-10)	7.27e-11 (-7.63e-10,9.09e-10)	-3.04e-10 (-7.18e-10,1.10e-10)	-2.93e-10 (-4.01e-10,-1.85e-10)
**Days 4 doses squared**	-1.62e-08 (-6.19e-08,2.95e-08)	-1.34e-08 (-4.20e-08,1.52e-08)	-2.54e-08 (-3.57e-08,-1.50e-08)	-5.46e-09 (-2.35e-08,1.26e-08)	1.56e-09 (-8.04e-09,1.12e-08)	4.21e-10 (-3.09e-09,3.93e-09)	3.22e-09 (-5.07e-09,1.15e-08)	-2.72e-10 (-4.09e-09,3.55e-09)	-1.14e-09 (-2.25e-09,-3.25e-11)

*Coefficients for COVID-19 hospitalization not shown, as interpretation is confounded by mandatory inpatient management of unvaccinated/partially-vaccinated patients with confirmed COVID-19 infection

Note: Regressions conducted using methods previously described by Bar-On et al[17] with time since last dose included as a continuous variable with both linear and quadratic terms. Positive coefficients for the linear terms (Days 2/3/4 doses) are indicative of rising IRRs and thus waning vaccine effectiveness.

© 2023 Tan WC et al. *JAMA Oncol.*

eTable 12: Person-Days and Incidence Rate/Million Person-Days by Time from Last Vaccine Dose

Delta Phase [a]												
	Person-Days			COVID-19 Incidence (Incidence Rate/Million Person-Days)			COVID-19 Hospitalization (Incidence Rate/Million Person-Days)			COVID-19 Severe Disease (Incidence Rate/Million Person-Days)		
	Active Treatment	Cancer Survivors	Control	Active Treatment	Cancer Survivors	Control	Active Treatment	Cancer Survivors	Control	Active Treatment	Cancer Survivors	Control
2 doses (8-59 days)	321,486	354,839	2,520,274	392	409	342	208	158	91	40	25	21
2 doses (60-149 days)	676,158	1,322,865	9,985,414	476	422	521	155	79	58	25	17	14
2 doses (>150 days)	346,839	998,446	7,762,367	649	477	914	205	104	127	29	26	31
3 doses (8-59 days)	348,181	1,124,914	7,385,459	184	167	162	46	34	15	6	4	2
3 doses (60-149 days)	77,004	282,635	1,823,839	65	60	73	39	4	7	0	0	1
Omicron Phase [b]												
	Person-Days			COVID-19 Incidence (Incidence Rate/Million Person-Days)			COVID-19 Hospitalization (Incidence Rate/Million Person-Days)			COVID-19 Severe Disease (Incidence Rate/Million Person-Days)		
	Active Treatment	Cancer Survivors	Control	Active Treatment	Cancer Survivors	Control	Active Treatment	Cancer Survivors	Control	Active Treatment	Cancer Survivors	Control
2 doses (8-59 days)	15,887	18,509	147,255	2455	1891	1514	567	648	374	189	324	68
2 doses (60-149 days)	151,589	163,174	1,257,130	2790	2623	2239	752	668	407	112	153	121
2 doses (>150 days)	299,813	385,790	4,388,534	2341	2323	2066	710	461	248	97	130	66
3 doses (8-59 days)	413,179	704,217	5,363,060	2002	1765	1558	322	121	72	24	26	12
3 doses (60-149 days)	1,152,800	2,606,521	18,745,706	1885	1965	1803	260	129	69	20	28	13

© 2023 Tan WC et al. *JAMA Oncol.*

3 doses (>150 days)	1,742,903	4,681,974	34,115,004	1551	1572	1341	231	106	55	36	17	10
4 doses (8-59 days)	197,556	561,875	3,765,443	1043	1086	914	106	69	50	20	12	6
4 doses (60-149 days)	225,623	720,423	4,584,378	1299	1366	1174	191	135	95	22	21	14

Note: Table supplementing IRR numbers presented in the main paper

© 2023 Tan WC et al. *JAMA Oncol.*
