# Supplementary material for: COVID-19 Severity and Waning Immunity After up to 4 mRNA Vaccine Doses in 73 608 Patients With Cancer and 621 475 Matched Controls in Singapore: A Nationwide Cohort Study
Source: JAMA Oncol. 2023 Jul 13;9(9):1221–9. doi: 10.1001/jamaoncol.2023.2271 (PMC10346511; doi:10.1001/jamaoncol.2023.2271)
Supplement: Supplement 2. — Data Sharing Statement [file jamaoncol-e232271-s002.pdf]

Data Sharing Statement

Tan. COVID-19 Severity and Waning Immunity After up to 4 mRNA Vaccine Doses in 73 608 Patients With Cancer and 621 475 Matched Controls in Singapore. *JAMA Oncol*. Published July 13, 2023. doi:10.1001/jamaoncol.2023.2271

Data
Data available: No

Additional Information
Explanation for why data not available: Requests for available data may be directed to the corresponding authors.
